# Ultrasound-responsive phosphorescence in aqueous solution enabled by microscale rigid framework engineering of carbon nanodots

**DOI:** 10.1038/s41377-025-01965-0

**Published:** 2025-09-11

**Authors:** Yachuan Liang, Haochun Shao, Kaikai Liu, Qing Cao, Sifan Zhang, Haiyan Wang, Liying Jiang, Chongxin Shan, Leman Kuang, Hui Jing

**Affiliations:** 1 School of Electronics and Information, Zhengzhou University of Light Industry, 450002 Zhengzhou, China; 2 Academy for Quantum Science and Technology, Zhengzhou University of Light Industry, 450002 Zhengzhou, China; 3 Henan Key Laboratory of Diamond Optoelectronic Material and Devices, School of Physics and Laboratory of Zhongyuan Light, Zhengzhou University, 450001 Zhengzhou, China; 4 College of Electrical and Information Engineering, Zhengzhou University of Light Industry, 450002 Zhengzhou, China; 5 Key Laboratory of Low-Dimensional Quantum Structures and Quantum Control of Ministry of Education, Department of Physics and Synergetic Innovation Center for Quantum Effects and Applications, Hunan Normal University, 410081 Changsha, China

**Keywords:** Nanoparticles, Quantum dots

## Abstract

Solid-state phosphorescent materials with stimulus-responsive properties have been widely developed for diverse applications. However, the task of generating excited states with long lifetimes in aqueous solution remains challenging due to the ultrafast deactivation of the triplet excitons and the difficulty in regulating stimulation sites in an aqueous environment. Additionally, most existing materials are primarily responsive to limited stimuli, such as light, oxygen, or temperature. Here, we present a microscale rigid framework engineering strategy that can be used to modulate the phosphorescence properties of carbon nanodots (CNDs), by brightening triplet excitons through ultrasound-enhanced rigidity in CNDs. Ultrasound-responsive phosphorescent CNDs with a lifetime of 1.25 seconds in an aqueous solution were achieved. The CNDs exhibit high sensitivity to surrounding ultrasound, showing a linear response to ultrasound exposure during the treatment period. The ultrasound-responsive phosphorescent CNDs demonstrate potential applications as sensing units in ultrasound radar detection and in vivo afterglow imaging.

## Abstract (web summary)

Ultrasound-responsive triplet excitons emission in CNDs with a tunable lifetime up to 1.25 s in aqueous solution via microscale rigid framework engineering strategy have been achieved.

## Graphical abstract

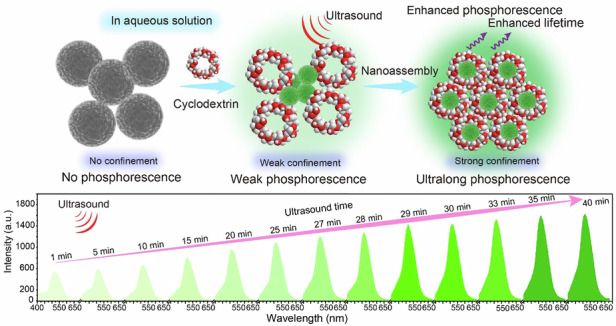

## Introduction

Excitons with long-lived excited states emit photons over extended durations, thereby improving signal-to-noise ratio and tissue penetration, which is crucial for non-invasive and deep-tissue imaging. Stimulus-responsive fluorescent materials play a significant role in optical sensing for detecting chemical and environmental changes, non-invasive diagnostics and real-time tracking, and data encryption through dynamic emission properties. In particular, stimulus-responsive room-temperature phosphorescent (RTP) materials have attracted considerable attention in these fields due to their unique long-lived excited state properties, which can eliminate the interference of background fluorescence, providing a novel solution for practical applications^1–8^. Background fluorescence typically exhibits an emission lifetime on the nanosecond scale, while the long-lived excited state properties of RTP materials enable them to be recorded in time-gated mode, thus avoiding interference from background fluorescence. This characteristic is particularly crucial in optical detection and imaging technologies as it allows for high-contrast signal detection in complex environments. However, the development of stimulus-responsive RTP materials remains in its infancy. Several key challenges impede progress: Firstly, long-lived triplet excitons are rapidly depleted through non-radiative relaxation pathways and quenched by oxygen. Secondly, managing both triplet excitons and stimulus-responsive sites concurrently is extremely intricate and arduous^9–16^. Thus, achieving long-lasting stimulus-responsive RTP materials remain a challenge.

RTP carbon nanodots (CNDs), a new generation of zero-dimensional long-lasting emissive carbon nanomaterials, have been widely applied in the fields of sensors, energy storage, bioimaging, and delay lighting. Given their unique properties, CNDs are regarded as promising candidates for constructing long-lasting emissive stimulus-responsive RTP materials^17–23^. Previous investigations on stimulus-responsive RTP CNDs indicate that they exhibit sensitivity to oxygen, temperature, and light exposure. Recent research has shown that by employing rational molecular design of the CNDs-based emitters and a two-component design strategy, some specific external stimulus can induce alterations in the photophysical properties. For example, thermal-responsive phosphorescence is realized by embedding either originally synthesized CNDs or thermal-treated CNDs into a polyvinylalcohol matrix through post-synthetic thermal annealing at high temperature. In addition, the luminescence lifetimes of the CNDs can be manipulated from the nanosecond level to the second level by introducing water, exhibiting high sensitivity to water^24–28^. A distinctive dynamic RTP is observed in the flexible solid films composed of luminescent CNDs and polypyrrolidone, enabling complete reversibility in light activation and thermal deactivation of phosphorescence. That is to say, regulating the microenvironment around CNDs can control their photophysical properties. However, the range of external stimuli that can elicit an optical response in phosphorescent materials are still relatively restricted compared to those materials that exhibit stimuli-responsive fluorescence^29–32^. Triplet excited state electrons are highly susceptible to oxygen, making phosphorescent CNDs effective for detecting dissolved oxygen in water. Due to their long-lived lifetimes, water-soluble phosphorescent CNDs have been implemented in time-gated bioimaging, displaying distinct advantages to function effectively even in the presence of a strong fluorescent background^30,33–35^. However, most stimulus-responsive phosphorescent materials operate primarily in solid-state forms, with few reports on stimulus-responsive phosphorescence in aqueous media. Therefore, the development of stimulus-responsive RTP CNDs with long lifetimes in water is of significant importance for their potential applications in a stimulus environment^32,36–40^.

Triplet electrons have longer lifetimes in the excited state, allowing competing photophysical processes, like fluorescence decay, non-radiative decay, and oxygen-induced interactions, to compete with phosphorescence for dominance^14,41–44^. To date, strategies such as crystal engineering, embedding in a rigid matrix, polymerization, and H-aggregation have been considered for obtaining efficient room temperature phosphorescence^1,6,45^. Among these strategies, the external microenvironment regulation strategy, which modulates the excited state properties of luminescent units, demonstrates a powerful capability to manufacture high-performance phosphorescent materials under varying environmental conditions. For example, confining CNDs through silica matrices can suppress the intramolecular motion of luminescent units and minimize the non-radiative decay of the triplet excitons^27,46^. In systems composed of energy donor molecules and energy acceptor molecules, photo-induced energy transfer and rigid environment enhancement have been reported to form persistent phosphorescent materials lasting for several hours. If external stimuli can control the rigid environment safeguarding triplet excitons, stimulus-responsive phosphorescent CNDs can be achieved. Thus, the external stimuli dismantle or establish a rigid microenvironment at a microscopic scale could offer a more straightforward approach to the development of stimulus-responsive phosphorescence CNDs. Compared to conventional stimuli like light or heat, ultrasound enables non-contact energy delivery with high spatial resolution, allowing modulation of rigid microenvironment without affecting emitter. With penetration depths exceeding 5 cm in organism, ultrasound overcomes the opacity limitations of photonic stimuli, making it ideal for stimulus source. In addition, the mechanical effect of ultrasound can dynamically regulate the rigid microenvironment at the material interface, which may achieve real-time phosphorescence intensity modulation.

Herein, we report ultrasound-responsive RTP CNDs achieved by constructing a microscale rigid framework through self-assembly of cyclodextrin in aqueous solution. The phosphorescence of the CNDs is not detectable without ultrasonic stimulation, due to the strong non-radiative recombination of triplet excitons. Following several minutes ultrasound treatment, the assembly process of cyclodextrin is completed in an aqueous solution. The CNDs are confined within the framework of cyclodextrin through hydrogen bonding interactions, resulting in enhanced RTP performance in aqueous solution, with RTP lifetime increase to 1.25 s. The ultrasound responsiveness of the CNDs is correlated with the degree of crystallinity of the cyclodextrin framework. Furthermore, the ultrasound-responsive multicolor afterglow was demonstrated in aqueous solution through the Förster resonant energy transfer process. As sensing units, the ultrasound-responsive RTP CNDs have shown potential applications in ultrasound radar detection and in vivo afterglow imaging.

## Results

**Fig. 1:**
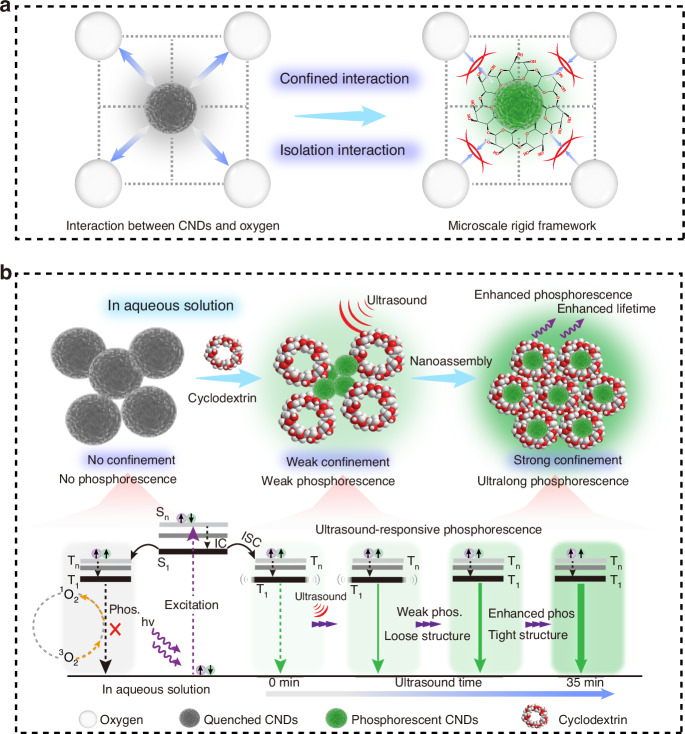
Schematic illustration of ultrasound-responsive phosphorescent CNDs. **a** Demonstration of ultrasound-responsive phosphorescent CNDs by microscale rigid framework engineering. **b** The mechanism diagram of ultrasound-responsive RTP CNDs, as well as related energy levels

**Fig. 2:**
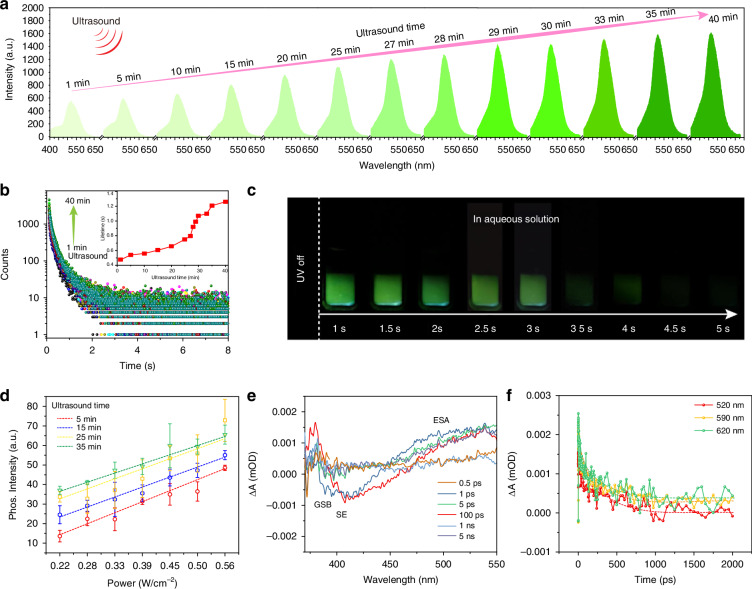
Photophysical properties of ultrasound-responsive CNDs. **a** Phosphorescence spectra of the CNDs treated at different ultrasound times. **b** Lifetime decay curves of the ultrasound-responsive CNDs at different ultrasound times, the inset is the lifetime under ultrasound treatment at different times. **c** The phosphorescent images of the ultrasound-responsive CNDs after treatment for 35 min. **d** The correlation between power density and phosphorescence intensity under ultrasound treatment at different times. **e** Femtosecond transient absorption (TA) spectra of ultrasound-responsive CNDs at indicated delay times from 0.5 ps to 5 ns. **f** Kinetic traces at different probe wavelengths of the ultrasound-responsive CNDs

**Fig. 3:**
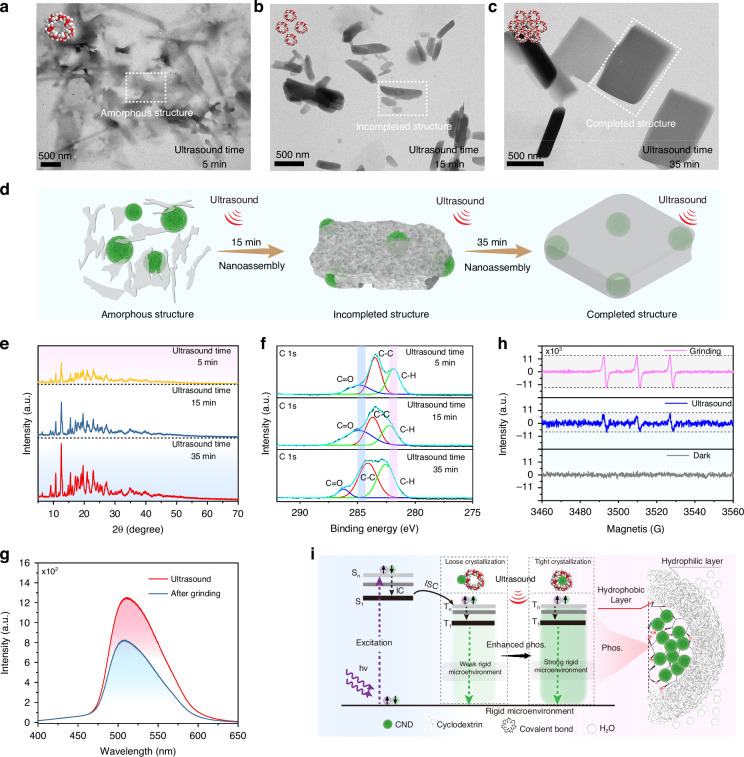
Mechanism investigation for ultrasound-responsive CNDs. **a-c** Transmission electron microscope (TEM) images of CNDs after being sonicated at different times. **d** Illustration of the nano-assembly process of CNDs and cyclodextrin under different ultrasonic times. **e** X-ray diffraction (XRD) of CNDs after being sonicated at different times. **f** C 1 s spectra of CNDs after being sonicated at different times. **g** Phosphorescence spectra of the ultrasound-responsive CNDs before and after grounding. **h** EPR spectra of ultrasound-responsive CNDs before and after grounding. **i** Proposed mechanism for ultrasound-responsive phosphorescence of CNDs

Due to weak spin-orbit coupling (SOC), as well as molecular motion and inhibitors such as oxygen and water in the surrounding environment, triplet excitons inevitably dissipate as illustrated in the left of Fig. 1a. It is worth noting that a variety of interactions, including hydrogen bonds, covalent bonds, etc., are used to restrict molecular motion^1^. Among them, constructing a microscale rigid framework through confined interactions can suppress non-radiative transitions from excited triplet states to ground states (Fig. 1a, Right). Considering that cyclodextrin has hydrophobic cavities and abundant hydrophilic hydroxyl groups, through self-assembly, the luminescent materials can be confined within the rigid hydrophobic cavity, thereby avoiding the influence of quenching agents in solution and inhibiting free molecular motion, leading to room temperature phosphorescence^47^. Meanwhile, the external hydrophilic groups can ensure water solubility. In addition, as the crystal structure of cyclodextrin gradually forms, the rigid degree of the environment around CNDs increases, and stimulus-responsive phosphorescence may be achieved. To verify our hypothesis, we chose phosphorescent CNDs as the emitter and studied their assembly process with cyclodextrin in aqueous solution under ultrasound stimulation. Firstly, the bare CNDs have been synthesized and characterized. Transmission electron microscopy (TEM) (Fig. S1) revealed that the average particle size of the CNDs without cyclodextrin was approximately 4 nm, which is consistent with our previous report^27^. The UV-vis absorption, photoluminescence, phosphorescence spectra and excitation-emission mapping of the bare CNDs were also recorded. The fluorescence and phosphorescence spectra of the CNDs are shown in Fig. S2 and the inset is the fluorescence and phosphorescence images. The fluorescence spectrum shows an emission peak at 445 nm, and no phosphorescence can be observed. The absorption spectrum of the CNDs displays two absorption peaks (Fig. S3a) at approximately 280 nm and 350 nm. These peaks are likely attributed to the π-π* and n-π* transitions. Additionally, considering the high degree of consistency between their excitation and absorption spectra, the n-π* transition may be responsible for their fluorescence emission. In addition, the lifetime decay curve of the CNDs, recorded at 445 nm, is presented in Fig. S3b. The corresponding fluorescence lifetime is 3.2 ns. The fluorescence excitation-emission contour of the CNDs is shown in Fig. S4. The fluorescence excitation wavelength is located in the range from 350 nm to 400 nm, and the optimum excitation wavelength is located at 375 nm. As expected, with the completion of the self-assembly of cyclodextrin, the confinement intensity around the CNDs increases, suppressing the non-radiative decay of triplet excitons, leading to ultralong phosphorescence in aqueous solution (Fig. 1b). In addition, the prepared CNDs also exhibit ultrasound responsiveness, making the system multifunctional. First, the photophysical properties of the ultrasound-responsive RTP CNDs were investigated (Fig. 2 and Figs. S5, S6). By studying the RTP performance of the CNDs and cyclodextrin assembly systems with different ratios, the optimal parameters for the ultrasound-responsive RTP CNDs are determined: 1.75 mL CNDs aqueous solution and 1.25 g cyclodextrin in deionized water (DI) to form aqueous solution. Figure 2 shows the phosphorescence spectra of the CNDs after being ultrasound treatment for different periods, featuring emission peaks located at 520 nm. Unexpectedly, as the ultrasound time increases, the phosphorescence intensity and phosphorescence lifetime of the CNDs gradually increases (Fig. 2a, b). After 35 min ultrasound treatment, the phosphorescence intensity reached its maximum, the maximum lifetime was 1.25 s, and the quantum yield (QY) reached 5.29%. The green phosphorescence in aqueous solution lasts for at least 5 s (Fig. 2c). The completion of the self-assembly of cyclodextrin after a longer period of ultrasound treatment, the surrounding rigidity may remain unchanged, and the change in phosphorescence intensity appears stable^48^. To verify the effect of ultrasound stimulation, the mixture of CNDs and cyclodextrin was stirred in water at room temperature for 20 min, and no visible RTP emission was observed (Fig. S7). The possible reason is that without ultrasound stimulation, CNDs molecules are not able to enter the hydrophobic cavity of cyclodextrin, and cyclodextrin is also not to complete self-assembly to form a rigid framework. The 3D phosphorescence spectrum shows a main emission band at approximately 520 nm under excitation from 200 to 400 nm (Fig. S8). Time-resolved emission spectroscopy (TRES) displays the phosphorescence wavelength and lifetime of the sample, as shown in Fig. S9. It was found that the profiles of the phosphorescence fixed with main peaks at 520 nm as time decay. In addition, the ultralong phosphorescence lifetime of the CNDs under ultrasound treatment endows them with a wide range of applications. Figure 2d shows the correlation between ultrasonic power density and phosphorescence intensity under ultrasound treatment at different times. At a specific ultrasound treatment time, there is a linear relationship between the two as the ultrasound power density increases. When the ultrasound time increases from 5 to 35 min, the slope of this linear dependence remains almost unchanged. In addition, at the same ultrasonic power density, the phosphorescence intensity of the CNDs increases with the increase of ultrasound time. The data exhibit an excellent linear correlation over this range, with a correlation coefficient (R²) of 0.985. Based on our measurements, the calculated detection limit of ultrasound power density is 8.2 mW cm^−^². The linear relationship between phosphorescence intensity and ultrasonic power density makes it promising for application in ultrasound quantification. The ΔE_ST_ of CNDs can be estimated by low-temperature fluorescence and phosphorescence spectra. In Fig. S10, CNDs exhibits two emission peaks at 405 nm and 530 nm in its low-temperature fluorescence and phosphorescence spectra at 77 K, and the ΔE_ST_ is calculated to be 0.72 eV. According to the following Eq. (1):

1 $${k}_{{ISC}}=\frac{2\pi }{{{\hbar }}}{\left|\left\langle S\left|{\hat{H}}_{{SOC}}\right|T\right\rangle \right|}^{2}\sqrt{\frac{\pi }{\lambda {K}_{B}T}}exp \left[-\frac{{\left(\Delta {E}_{{ST}}-\lambda \right)}^{2}}{4\lambda {K}_{B}T}\right]$$

the ${k}_{{ISC}}$ can be seriously influenced by the singlet-triplet splitting energy^1^. Thus, the ISC process for as-prepared CNDs was easy to occur due to the small energy gap Δ*E*_*ST*_. In addition, the temperature-dependent decay curves and phosphorescence spectra of CNDs were recorded from 77 to 297 K, as shown in Fig. S11. The lifetimes and phosphorescence intensity decrease with increasing temperature, which is a characteristic property of phosphorescent materials. To further investigate the dynamic process of charge carriers generated after excitation, the excited state spectra of CNDs were studied through ultrafast femtosecond transient absorption (TA) spectroscopy. Figure 2e shows the TA spectra of CNDs with probe wavelengths ranging from 340 to 550 nm and delay times ranging from 0.5 ps to 5 ns. The strong negative characteristics from 350 to 410 nm correspond to ground-state bleaching (GSB) and stimulated emission (SE). The negative peaks centered at around 380 nm gradually increase within 100 ps, consistent with steady-state fluorescence and absorption spectra (Figs. S12 and S13). Figure 2f also shows the kinetics of carriers at different wavelengths as a function of delay time. The evolution of the carriers at different excitation levels can be inferred. The generated hot carriers are relaxed to a free exciton (FE) state through internal conversion (IC), and then further relaxed to the first singlet state due to strong electron–phonon coupling^49^. The evolution of the singlet state can be directly detected through the decay curves at 410 nm. Subsequently, the singlet state is transformed into long-lived species through an intersystem crossing (ISC) process, characterized by a long decay curve at 520 nm (Fig. S14).It is known that ultrasound can promote collisions and interactions between molecules, thereby facilitating the self-assembly process between molecules. In order to reveal the reasons for ultrasound-responsive phosphorescence, the morphology of the assembled system after different ultrasound times were characterized by transmission electron microscopy (TEM). An amorphous sheet-like structure gradually formed when the mixture was sonicated for 5 min (Fig. 3a). At this point, the self-assembly of the cyclodextrin is in its initial stage. When the mixture was sonicated for 15 min, irregular block structures gradually formed (Fig. 3b). The self-assembly of the cyclodextrin is nearing completion. Finally, after 35 min of ultrasound, the cyclodextrin forms a complete crystal structure (Fig. 3c). Ultrasonic treatment can promote the aggregation of cyclodextrin monomers, form nanostructured domains through hydrogen bonding, and further trigger the polymerization of more monomers, forming a microscale rigid framework (Fig. 3d). As for why phosphorescent materials are sensitive to stimuli, it can be noted that different structures of phosphorescent materials formed under different ultrasound times during self-assembly exhibit different stimulus-responsive behaviors. Given that cyclodextrin has a hydrophobic cavity, we speculate that the ultrasound-responsive RTP emission in aqueous solution may originate from the inclusion interaction and intermolecular hydrogen bonding interactions between CNDs and cyclodextrin that vary with ultrasound times^7,50^. As is well known, when CNDs enters their hydrophobic cavities, cyclodextrin can provide a microscale rigid framework with different binding strengths for CNDs in different self-assembly states, which can lead to the ultrasound-responsive characteristics of CNDs. To validate this hypothesis, the powder X-ray diffraction (XRD) was performed on the powder samples treated with different ultrasound times. As shown in Fig. 3e, the XRD pattern shows that the peak intensity of the sample increases with the increase of ultrasound time, indicating a significant increase in crystallinity with the completion of cyclodextrin self-assembly, which is consistent with the TEM results. As the crystallinity increases, the rigid environment around CNDs increases, which can reduce non-radiative transition. To determine the confinement relationship between CNDs and cyclodextrin under different ultrasound times, the X-ray photoelectron spectroscopy (XPS) spectra of CNDs (Fig. 3f), prepared using different ultrasound times, are analyzed. The comparison of high-resolution C 1s spectra shows that small shifts are observed with increasing ultrasound time, indicating that the cyclodextrin framework has caused changes in the chemical environment around the CNDs. Through the analysis of these three high-resolution spectra, no new covalent bonds were observed, indicating that the shift is attributed to the spatial confinement effect of the cyclodextrin framework. Specifically, the peak corresponding to the C = O in the samples subjected to 5 min and 35 min ultrasound treatments was initially observed at 288.6 eV and shifted to a higher binding energy of 287.4 eV. This movement may be attributed to the confined interaction formed between the CNDs and the cyclodextrin framework. From the Fourier transform infrared spectroscopy (FTIR) spectra (Fig. S15), it can be observed that the intensity of the stretching vibration peak gradually increases with the increase of ultrasound time, which may be due to the interaction between CNDs and cyclodextrin, leading to the enhancement of valence bond stretching vibration. Moreover, the rate equation of population over time for triplet states is described as follows:

2 $$\frac{{{d}}\left[{T}_{1}\right]}{{{d}}t}={k}_{{ISC}}\left[{S}_{1}\right]-{k}_{p}\left[{T}_{1}\right]-\sum _{i}{k}_{{nr}}^{{T}_{1}\to {S}_{0},i}\left[{T}_{1}\right]$$

where [${T}_{1}$] is the population of excitons in the excited triplet state, [${S}_{1}$] is the population of excitons in the excited singlet state, and ${k}_{{ISC}}$ is the intersystem crossing rate constant. $k$_*p*_ is the phosphorescent radiative transition rate constant, ${k}_{{nr}}^{{T}_{1}\to {S}_{0},i}$ represents various non-radiative transition rate constants from the T_1_ state to the ground state S_0_. It can be found that the larger the non-radiative transition rate constant, the faster the decrease in [${{\rm{T}}}_{1}$] over time, and the corresponding decrease in phosphorescence intensity. In addition, Eq. (3) of the phosphorescence quantum yield is as follows:

3 $${{\phi }}_{P}=\frac{{k}_{p}}{{k}_{p}+\sum _{i}{k}_{{nr}}^{{T}_{1}\to {S}_{0},i}}$$

which reflects the competitive relationship between phosphorescent radiative transitions and non-radiative transitions. When the non-radiative transition rate constants ${k}_{{nr}}^{{T}_{1}\to {S}_{0},i}$ increases, the phosphorescence quantum yield ${{\phi }}_{P}$ decreases, leading to a decrease in phosphorescence intensity. The radiative and non-radiative transition rates under different ultrasound times can be calculated as follows:

4 $${k}_{p}={{\phi }}_{p}/{{\tau }}_{ph{os}}$$

5 $${k}_{{nr}}=(1-{{\phi }}_{p})/{{\tau }}_{ph{os}}$$

where *k*_*p*_ and *k*_*nr*_ represent the radiative and non-radiative transition rates, *τ*_*phos*_ denotes the phosphorescence lifetime, and *ϕ*_*p*_ is the phosphorescence efficiency^1,39,49^. Thus, the radiative and non-radiative transition rates can be determined from the measured phosphorescence efficiency and phosphorescence lifetime of the ultrasound-responsive CNDs. The dynamic photophysical parameters are shown in Table S1. It can be found that the radiative transition rate of the ultrasound-responsive CNDs increases with increasing ultrasound time due to the enhanced confinement effect of the cyclodextrin framework, which makes the CNDs ultrasound-responsive phosphorescence in aqueous solution. To further elucidate the mechanism behind the ultrasound-responsive phosphorescence, we investigated the phosphorescent behavior of the solid-state CNDs by applying mechanical force. As shown in Fig. 3g and Fig. S16, the phosphorescence of the grinding CNDs powder was diminished, indicating that the decrease in crystallinity led to a reduction in the rigid environment surrounding the CNDs, ultimately resulting in weakened phosphorescence. This phenomenon further indicates that the crystallinity of the cyclodextrin framework plays a crucial role in ultrasound-responsive RTP emission. Electron paramagnetic resonance (EPR) spectroscopy was conducted on CNDs before and after grinding, as shown in Fig. 3h. Singlet oxygen can be captured by specific probes to generate free radicals, thereby producing an EPR signal. Compared with the original CNDs, the signal intensity of the grinding CNDs is higher, which may be due to mechanical force disrupting the cyclodextrin framework structure, leading to more contact with oxygen. In other words, the cyclodextrin framework can, to a certain extent, prevent the interaction between triplet excitons and oxygen. To further demonstrate the confinement relationship between CNDs and cyclodextrin, as a control experiment, weak RTP emission was observed when CNDs and cyclodextrin were mixed through simple grinding (Fig. S17). This may be due to the weak confinement effect caused by hydrogen bonding between CNDs and cyclodextrin, which limits the molecular vibration of CNDs^47,50^. That is to say, simple grinding cannot allow CNDs to enter the hydrophobic cavity to restrict their molecular movement, and the hydrogen bonding alone is not sufficient to ensure the ultralong phosphorescence emission of CNDs. Overall, we propose a reasonable mechanism for the ultrasound-responsive phosphorescence of CNDs. As shown in Fig. 3i, thanks to the hydrophobic inner cavity and hydrophilic outer cavity of cyclodextrin, under ultrasound treatment, cyclodextrin gradually self-assembles into a rigid framework, with CNDs entering its hydrophobic cavity. The progressively complete rigid framework provides a rigid environment to restrict the molecular motion of the CNDs and reduce the quenching by surrounding oxygen. Consequently, the triplet excitons are stabilized, and non-radiative transitions are effectively suppressed, resulting in ultralong phosphorescence^27,33^. Meanwhile, cyclodextrin with ultrasound-enhanced self-assembly properties plays a crucial role in stimulus response. As the cyclodextrin self-assembly is completed, the rigid framework offers a variable protective barrier for the triplet excitons, thereby endowing CNDs with ultrasound-responsive characteristics.

To validate our hypothesis and gain further insight into the underlying mechanisms, theoretical calculations using first-principles time-dependent density functional theory (TD-DFT) were performed on both bare CNDs and ultrasound-responsive CNDs in singlet and triplet excited states. As shown in Fig. S18, the energy gap between the singlet and triplet states of CNDs was small, facilitating the intersystem crossing (ISC) process. The spin-orbit couplings (ξ) and the nature of the transition orbitals for both bare CNDs and ultrasound-responsive CNDs are depicted in Fig. S18. Notably, the spin-orbit coupling (SOC) ξ (S_1_, T_n_) of the ultrasound-responsive CNDs (11.87 cm^−^¹) was significantly larger than that of bare CNDs (2.93 cm^−^¹), which is favorable for phosphorescence. This suggests that during the self-assembly process of cyclodextrin, hydrogen bonds effectively anchor the CNDs, promoting ISC from singlet to triplet states and thereby leading to efficient phosphorescence. Additionally, it was observed that the ultrasound-responsive CNDs exhibited an increase in radiative transitions and a decrease in non-radiative decay rates with prolonged ultrasound exposure (Table S1), contributing to the extended emission lifetime of the ultrasound-responsive CNDs. Based on these results, we conclude that highly efficient ultrasound-responsive phosphorescence was ascribed to suppressing the non-radiative transition and populating triplet excitons through self-assembly of cyclodextrin around CNDs.

**Fig. 4:**
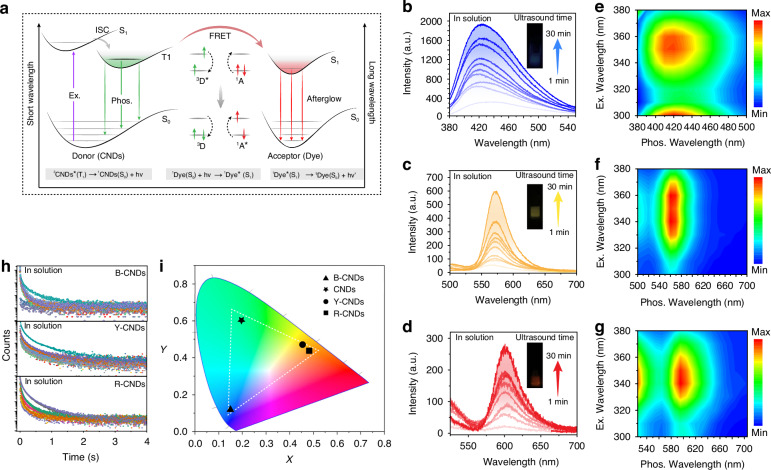
Photophysical properties of B-CNDs, Y-CNDs and R-CNDs. **a** Proposed mechanism of energy transfer from donor (phosphorescence CND) to the acceptor (dyes). **b-d** Phosphorescence spectra of the B-CNDs, Y-CNDs and R-CNDs at different ultrasound times. **e-g** Excitation-phosphorescence mapping of B-CNDs, Y-CNDs and R-CNDs. **h** Lifetime decay of the B-CNDs, Y-CNDs and R-CNDs at different ultrasound times. **i** CIE 1931 chromaticity diagram of the afterglow of B-CNDs, CNDs, Y-CNDs and R-CNDs

To demonstrate the universality of our approach, a series of new CNDs were mixed with the cyclodextrin matrix, which was named B-CNDs. In addition, Förster resonance energy transfer (FRET) has been proven to be the most accessible strategy to manufacture multi-color afterglow materials by combining the advantages of long-lived phosphorescent donors and fluorescent acceptors. Considering that cyclodextrin can effectively isolate fluorescent dyes and CNDs, it is feasible to adjust the afterglow color through the reabsorption of radiation energy transfer. The prerequisite for energy transfer is the overlap between the emission spectrum of the energy donor and the absorption spectrum of the energy acceptor. As shown in Figs. 4a and S19, the absorption spectra of Rhodamine 6 G and Rhodamine B overlap significantly with the phosphorescence emission spectra of CNDs, which comprise a potentially efficient FRET pair. The FRET process can be described according to the equations of the energy transfer as follows:

6 $${}^{3}{\rm{CNDs}}^{* }({{\rm{T}}}_{1})\to {}^{1}{\rm{CNDs}}({{\rm{S}}}_{0})+hv$$

7 $${}^{1}{\rm{Dye}}({{\rm{S}}}_{0})+hv\to {}^{1}{\rm{Dye}}^{* }({{\rm{S}}}_{1})$$

8 $${\scriptstyle{1}\atop}{\rm{Dye}}^{*}({{\rm{S}}}_{1})\to{\scriptstyle{1}\atop}{\rm{Dye}}({{\rm{S}}}_{0})+hv{{\mbox{'}}}$$

Where ^3^CNDs^*^(T_1_) and ^1^CNDs(S_0_) are triplet excited state and singlet ground state of the RTP CNDs; ^1^Dye ^*^(S_1_) and ^1^Dye (S_0_) are single excited state and singlet ground state of dye; *h* is Planck constant and *v* and *v’* are emitted photons with different frequencies^1^. Therefore, Rhodamine 6G and Rhodamine B were chosen as energy acceptors to construct luminescent complexes and named as Y-CNDs and R-CNDs, respectively. Impressively, thanks to the structural limitations and hydrogen bonding interactions of cyclodextrin, these complexes can achieve multi-color afterglows of blue, yellow, and red after turning off the UV lamp. In particular, the phosphorescence spectra of B-CNDs, Y-CNDs and R-CNDs exhibit ultrasound-responsive characteristics, with their maximum emission peaks located at 425, 575, and 600 nm (Fig. 4b–d), respectively. In addition, as the excitation wavelength changes, the profile of the phosphorescence spectra remains unchanged (Fig. 4e–g). After optimization, B-CNDs, Y-CNDs and R-CNDs show the longest phosphorescence lifetimes of 993 ms, 883 ms, and 632 ms (Fig. 4h), respectively. Meanwhile, the CIE coordinates corresponding to B-CNDs, CNDs, Y-CNDs and R-CNDs are (0.14, 0.10), (0.21, 0.49), (0.45, 0.50) and (0.47, 0.49), spanning a large emission color gamut (Fig. 4i). In addition, in order to further evaluate the universality of microscale rigid framework engineering strategy, cucurbituril was selected as confined matrix. Upon encapsulation of the CNDs into cucurbituril matrix, we observed no enhancement of phosphorescence intensity under ultrasound treatment (Fig. S20), which suggests that the unique cavity environment of cyclodextrin molecules may play a critical role in facilitating triplet state stabilization. The reversibility is a critical feature for practical applications. So, a series of cycle experiments were carried out to evaluate the reversibility of the ultrasound-induced RTP effect. Specifically, we subjected the ultrasound-responsive CNDs to mechanical stress (Fig. S21a), the phosphorescence emission peak at 520 nm shows a significant decrease in intensity. Subsequently, the CNDs subjected to stress were exposed to ultrasound treatment, and a recovery in phosphorescence intensity was observed. To systematically verify the ultrasound-responsive reversibility, Fig. S21b displays cyclic testing where samples undergo five stress-ultrasound recovery cycles. Remarkably, the phosphorescence intensity maintains good consistency across all cycles without significant degradation, which not only confirms the reversible characteristics of the phosphorescence but also highlights its promising applicability in ultrasound sensing devices. The stability of CNDs is a key factor for the practical applications. Under continuous exposure to UV light for 10 min, the phosphorescence intensity only showed a slight decrease, as shown in Fig. S22, indicating that the prepared CNDs have excellent photostability. In addition, the photostability of the CNDs was also evaluated by continuously irradiating the sample with a 350 nm light source for up to 80 min (Fig. S23a), and the phosphorescence intensity remained above 90% of its initial value, demonstrating good resistance to photobleaching. In addition, the CNDs were stored at room temperature for a period of 10 days to assess the temporal stability. The phosphorescence intensity was recorded at regular intervals. The results showed minimal change in the emission intensity over time, indicating excellent temporal stability (Fig. S23b). These results indicate that the prepared ultrasound-responsive CNDs exhibit high stability, making them suitable for practical applications. To our knowledge, compared to reported CND-based phosphorescent materials, these composites that exhibit both long-lifetime and ultrasound-responsive characteristics under environmental conditions are the first report.

## Discussion

**Fig. 5:**
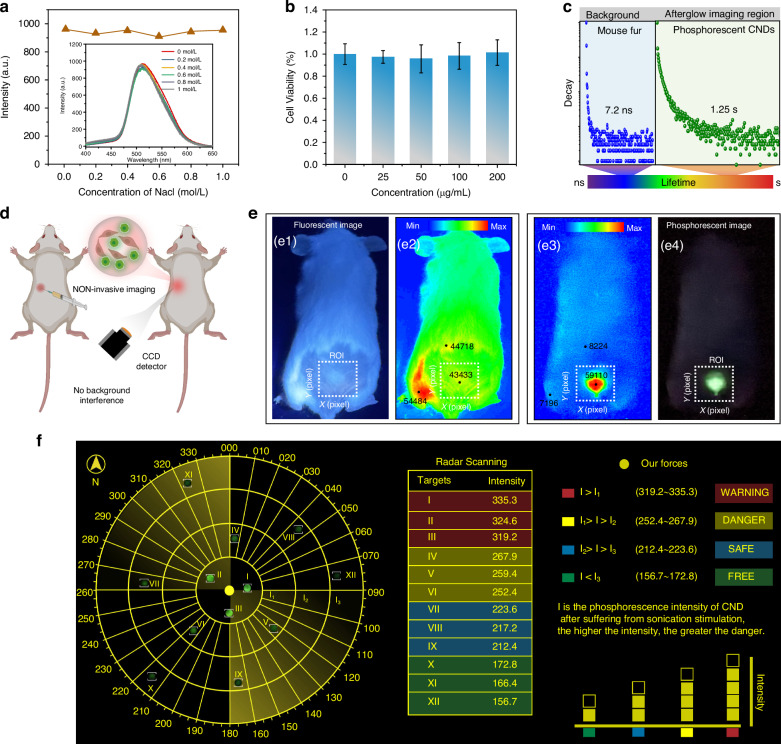
Demonstration of ultrasound-responsive CNDs for afterglow imaging and ultrasound detection. **a** Phosphorescence intensity of the CNDs under NaCl aqueous solution with different concentrations. **b** Cytotoxicity assay of the CNDs. **c** Illustration of time-gated bioimaging. **d** Schematic illustration of in situ activation and detection of phosphorescence of the CNDs in living mice. **e** In vivo fluorescence and phosphorescence images of mice with the subcutaneous implantation of CNDs. **f** Demonstration of radar detection mimicry by employing CNDs as a response source

To demonstrate the potential of as-prepared material in biomedical applications, the stability of the CNDs was evaluated. The relationship between the afterglow intensity and the concentration of NaCl aqueous solution was recorded, and even when the concentration reached 1 mol L^−1^, the afterglow could still be maintained (Fig. 5a), indicating good stability in NaCl aqueous solution. The corresponding afterglow spectra are shown in the inset of Fig. 5a. Before biological application, the biocompatibility of CNDs was assessed using AML12 cells in a CCK-8 assay, as shown in Fig. 5b. A series of CNDs concentrations ranging from 0 to 200 μg mL^−1^ exhibited negligible cytotoxicity. Even at a concentration of 200 μg mL^−1^ of CNDs, the cell viability remained above 90% after incubating for 24 h. The results suggest that CNDs have low cytotoxicity and high stability, which is beneficial for phosphorescent bioimaging. Thanks to the merit of low toxicity and long-lived afterglow emission, CNDs can be used for in vivo afterglow bioimaging based on time-gating technology. The in vivo afterglow photo was taken after removing the excitation source. Mouse fur (background emission) and CNDs have different lifetimes of 7.2 ns and 1.25 s, respectively. Therefore, the signal collected after 7.2 ns comes from CNDs and the background emission of mouse fur has been eliminated (Fig. 5c). To demonstrate the potential of employing CNDs for in vivo phosphorescent imaging, CNDs were subcutaneously injected into the region of interest (ROI) of live nude mice. Images before and after ultraviolet (UV) irradiation were captured using a charge-coupled device equipped with a regular smartphone, as shown in Fig. 5d. In the fluorescence imaging mode, the mouse fur exhibited strong background fluorescence, making it difficult to observe the imaging signal (Fig. 5e1). To assess the imaging quality, the intensity distribution of the images was extracted and analyzed. As shown in Fig. 5e2, the intensity details of each pixel were depicted from the image, and the signal intensity of the imaging agent (43433) in the ROI was much lower than that of the background fluorescence (54484). In the phosphorescent imaging mode, a strong signal in the ROI can be observed, which was much higher than that of the dark areas. Through the intensity analysis of the image, the signal intensity of the imaging agent (59110) within the ROI was much greater than the intensity of the background fluorescence (8224), indicating that in vivo phosphorescent imaging can effectively eliminate the interference of auto-fluorescence. The signal-to-noise ratio was calculated based on the ratio of the luminescence intensity in the region of interest to that in the surrounding background of the mouse fur. The mouse fur background emission intensity is measured as 1799, and the luminescence intensity in the region of interest is measured as 60138; the signal-to-noise ratio for the afterglow in vivo imaging is as high as 15 dB, which confirms the potential of our CNDs for high-contrast in vivo imaging applications. Considering the ultrasound-responsive characteristics of CNDs, we further envision CNDs as a sensing unit for demonstrating radar all-round detection. Here, we conceptualize a radar system that uses ultrasound-responsive phosphorescent CNDs, capable of assessing the threat level of targets based on phosphorescent intensity. The radar scan is used to display the distribution of targets, with increasing phosphorescent intensity as targets approach our force. The table, on the other hand, is used to categorize the threat level of targets based on phosphorescent intensity (Fig. 5f). Such a system may be employed for security monitoring, military applications, or other scenarios requiring rapid assessment and response to potential threats.

In summary, we designed and demonstrated ultrasound-responsive RTP CNDs with an ultralong lifetime of 1.25 s through microscale rigid framework engineering. In this system, ultrasound facilitates the self-assembly of cyclodextrin into a rigid framework, which effectively suppress non-radiative transitions and avoid quenching agents, resulting in ultralong phosphorescence of CNDs in aqueous solution. In principle, stimuli-responsive phosphorescent materials can be achieved, if external stimuli can induce to form a rigid environment surrounding the luminescent component, and cut off the interaction between the luminescent component and the external quenching agents. Due to the high sensitivity to ultrasound in aqueous environments, they show promising applications in afterglow imaging and ultrasound detection.

## Materials and methods

### Materials

Ethylenediamine (EDA) (purity > 99%), phosphoric acid (purity > 95%), Tetraethoxysilane (purity > 99%), ammonia, Cyclodextrin (purity > 95%) and deionized water. All the chemicals were purchased from Macklin Chemistry Co. Ltd. (Shanghai, China). Note that all the chemicals used in this work were analytical grade without further purification.

### Synthesis of CNDs solution

Firstly, 1.0 mL of EDA solution was dissolved in 15 mL of deionized water, and then 2 mL of phosphoric acid was added to the EDA aqueous solution with stirring for 5 min. The formed transparent solution was then heated in a microwave oven (750 W) for 120 s. And then 20 mL deionized water was added to the above sample when the sample cooled down to room temperature, and the light yellow solution was obtained. The aqueous solution was centrifuged for 10 min in order to remove sediment. The supernatant was filtered through 0.22 μm membrane and the supernatant was collected.

### Synthesis of ultrasound-responsive CNDs

Disperse 1.75 ml of CNDs into 15 ml of deionized water, add 1.25 g of cyclodextrin, stir for 10 min, and then put it into an ultrasonic generator. Under different ultrasonic durations, the CNDs aqueous solution has ultrasonic responsive properties.

### Cellular Toxicity Test

In order to assess cellular toxicity, AML12 cells (1000 cells 200 μL^−1^) were cultured in 96-well plates in an incubator for 24 h (37 °C, 5% CO_2_). After that, the cultured medium was replaced by fresh medium, which contained the CNDs with different concentrations (0, 25, 50, 100 and 200 μg mL^−1^). The microplate reader was used to monitor absorbance at 490 nm and the corresponding cell viability was evaluated according to the following equation:

$${\rm{Cell\; Viability}}( \% )={{\rm{OD}}}_{{\rm{Treated}}}/{{\rm{OD}}}_{{\rm{Control}}}$$

### Quantum yield measurement

The quantum yield (QY) of the sample was measured to be 5.29%. The measurement was carried out using an FLS1000 spectrometer equipped with a calibrated integrating sphere. The QY was calculated using the following formula:

$${\rm{\eta }}=\frac{\varepsilon }{\alpha }=\frac{{\int }_{390}^{750}{L}_{{emission}}}{\int {E}_{{reference}}-\int {{\rm{E}}}_{{\rm{sample}}}}=\frac{{number\; of\; emitted\; photons}}{{number\; of\; absorbed\; photons}}$$

In this equation, *η* represents the quantum yield (QY), *ε* denotes the number of emitted photons from the sample that are captured by the integrating sphere over the 390–750 nm wavelength range, and α signifies the number of photons absorbed by the sample. *L*_emission_ indicates the net phosphorescent light emitted by the sample. *E*_reference_ corresponds to the absorption spectrum of the reference material inside the sphere, while *E*_sample_ refers to the combined absorption spectrum of both the sample and the reference. Based on this equation, the QY of the ultrasound-responsive CNDs was calculated to be 5.29%.

### Transient absorption measurement

Ultrafast transient absorption measurements were performed based on a Ti:sapphire laser (Spectra-Physics, Spitfire ACE, 800 nm, 4.5 mJ pulse^−1^, full width half maximum (fwhm) 35 fs, 1 kHz). In this experiment, pulses with a wavelength of 400 nm were used as a pump pulse.). The white-light probe was generated by focusing the fundamental laser output on a CaF_2_ window. The pump pulses were chopped to 25 Hz through a synchronized chopper (Newport, Model 3502). The probe pulse was focused into an optical fiber that is coupled to a spectrometer (Ocean Optics, QE PRO) after passing through the sample. The CNDs were placed in a 2 mm optical length quartz cuvette. The group velocity dispersion effect of the solvent was corrected by a chirp program.

### Characterization

The fluorescence and phosphorescence spectra were collected using the Hitachi F-7100 spectrophotometer and FLS1000 spectrometer. TEM images were recorded on JSM-6700F transmission electron microscope. The lifetimes of the CNDs were measured by time-correlated single photon counting on a FLS1000 spectrometer with excitation of 350 nm at room temperature. X-ray diffraction (XRD) patterns were obtained on X’Pert Pro diffractometer. The X-ray photoelectron spectra (XPS) were recorded using PERKIN ELMZR PHI 3056 X-ray photoelectron spectroscope.

## Supplementary information

SUPPLEMENTAL MATERIAL
