# Supplementary material for: Ultrasound-responsive phosphorescence in aqueous solution enabled by microscale rigid framework engineering of carbon nanodots
Source: Light Sci Appl. 2025 Sep 11;14:316. doi: 10.1038/s41377-025-01965-0 (PMC12426232; doi:10.1038/s41377-025-01965-0)
Supplement: Supplementary file 1 — SUPPLEMENTAL MATERIAL [file 41377_2025_1965_MOESM1_ESM.doc]

**Supporting Information**

**Ultrasound-Responsive Phosphorescence in Aqueous Solution Enabled by Microscale Rigid Framework Engineering of Carbon Nanodots**

Ya-Chuan Liang^1,2^, Hao-Chun Shao^1^, Kai-Kai Liu^3✉^, Qing Cao^3^, Si-Fan Zhang^4^, Hai-Yan Wang^1,2^, Li-Ying Jiang^1,2✉^, Chong-Xin Shan^3^, Le-Man Kuang^2,5^, Hui Jing^2,5✉^

^1^ School of Electronics and Information, Zhengzhou University of Light Industry, Zhengzhou 450002, China.

^2^ Academy for Quantum Science and Technology, Zhengzhou University of Light Industry, Zhengzhou 450002, China

^3^ Henan Key Laboratory of Diamond Optoelectronic Material and Devices, School of Physics and Laboratory of Zhongyuan Light, Zhengzhou University, Zhengzhou 450001, China

^4^ College of Electrical and Information Engineering, Zhengzhou University of Light Industry, Zhengzhou 450002, China

^5^ Key Laboratory of Low-Dimensional Quantum Structures and Quantum Control of Ministry of Education, Department of Physics and Synergetic Innovation Center for Quantum Effects and Applications, Hunan Normal University, Changsha 410081, China.

^✉^e-mail: [liukaikai@zzu.edu.cn](mailto:liukaikai@zzu.edu.cn) (Kai-Kai Liu); jinghui@hunnu.edu.cn (Hui Jing)


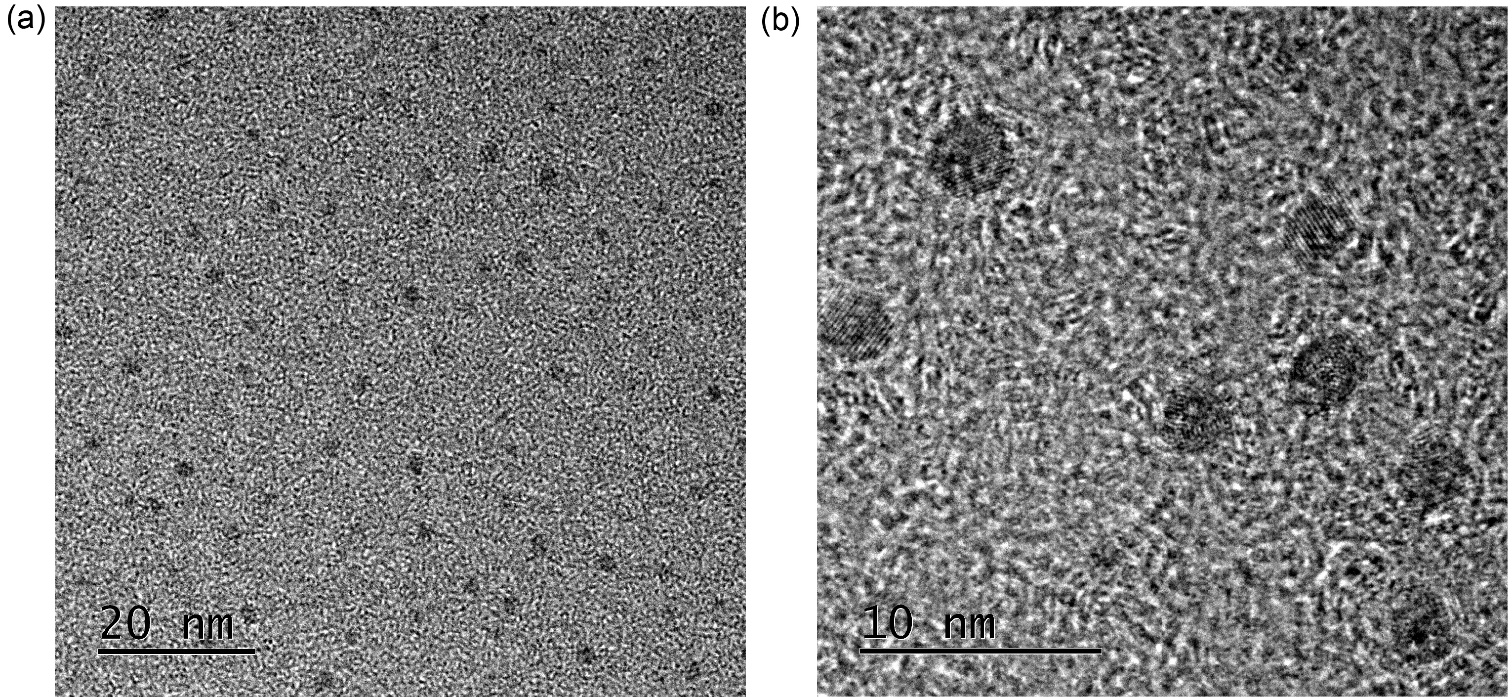


**Fig. S1** **a-b** TEM images of the CNDs without cyclodextrin.


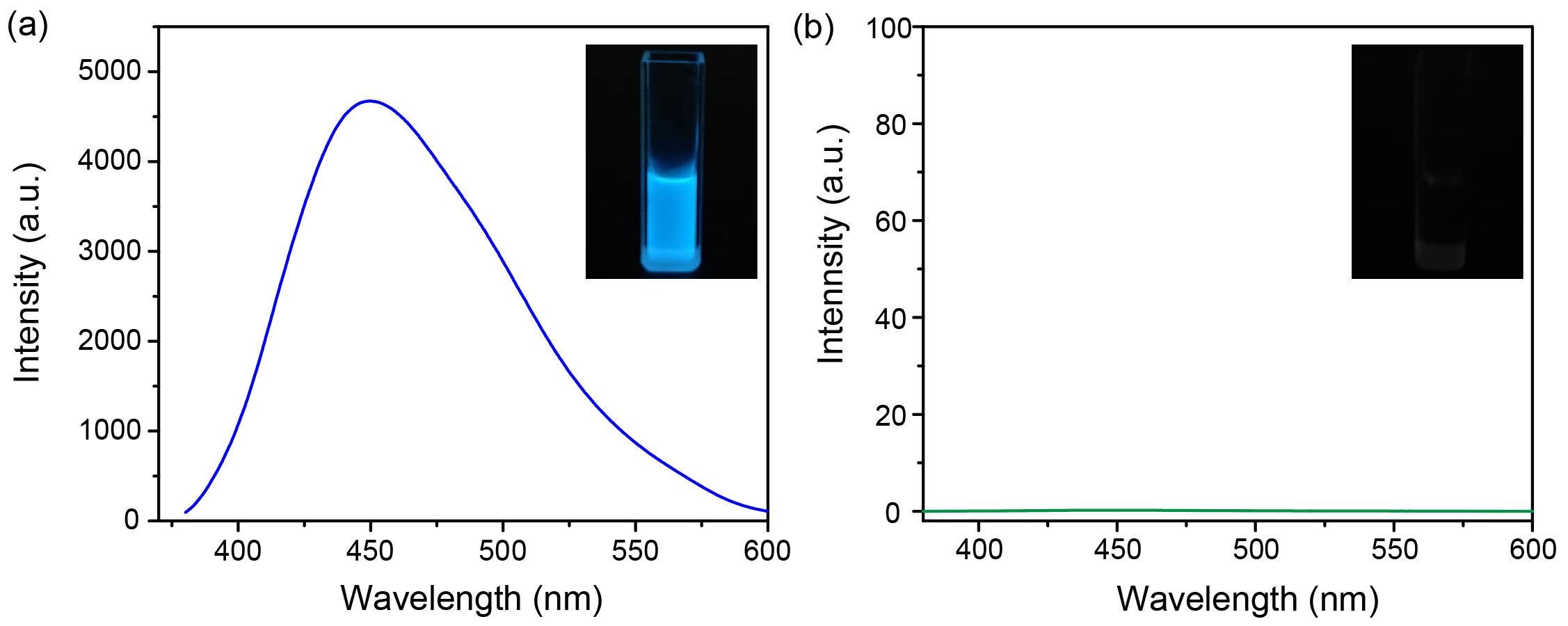


**Fig. S2** **a** The fluorescence spectrum of the CNDs (Inset: fluorescence image). **b** The phosphorescence spectrum of the CNDs. (Inset: phosphorescence image)


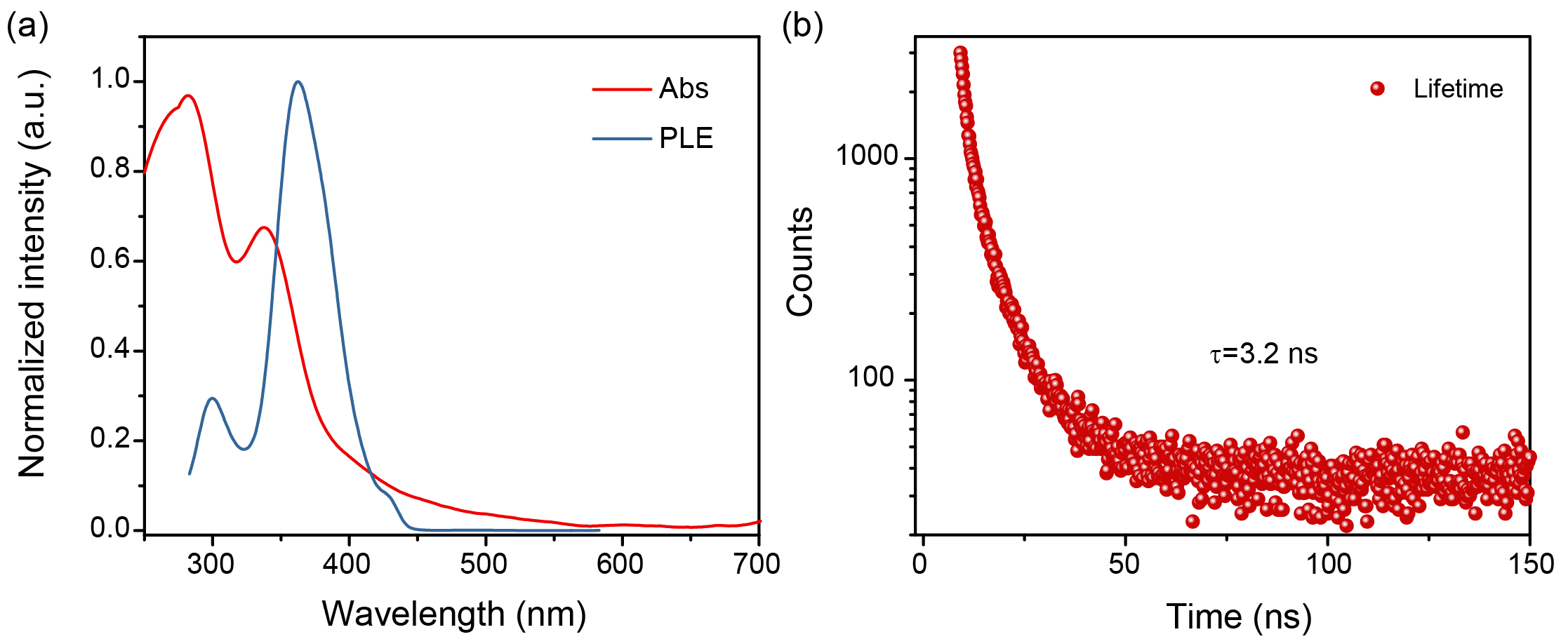


**Fig. S3** **a** Absorption spectrum and excitation spectrum of the CNDs. **b** The lifetime decay plots of the CNDs.


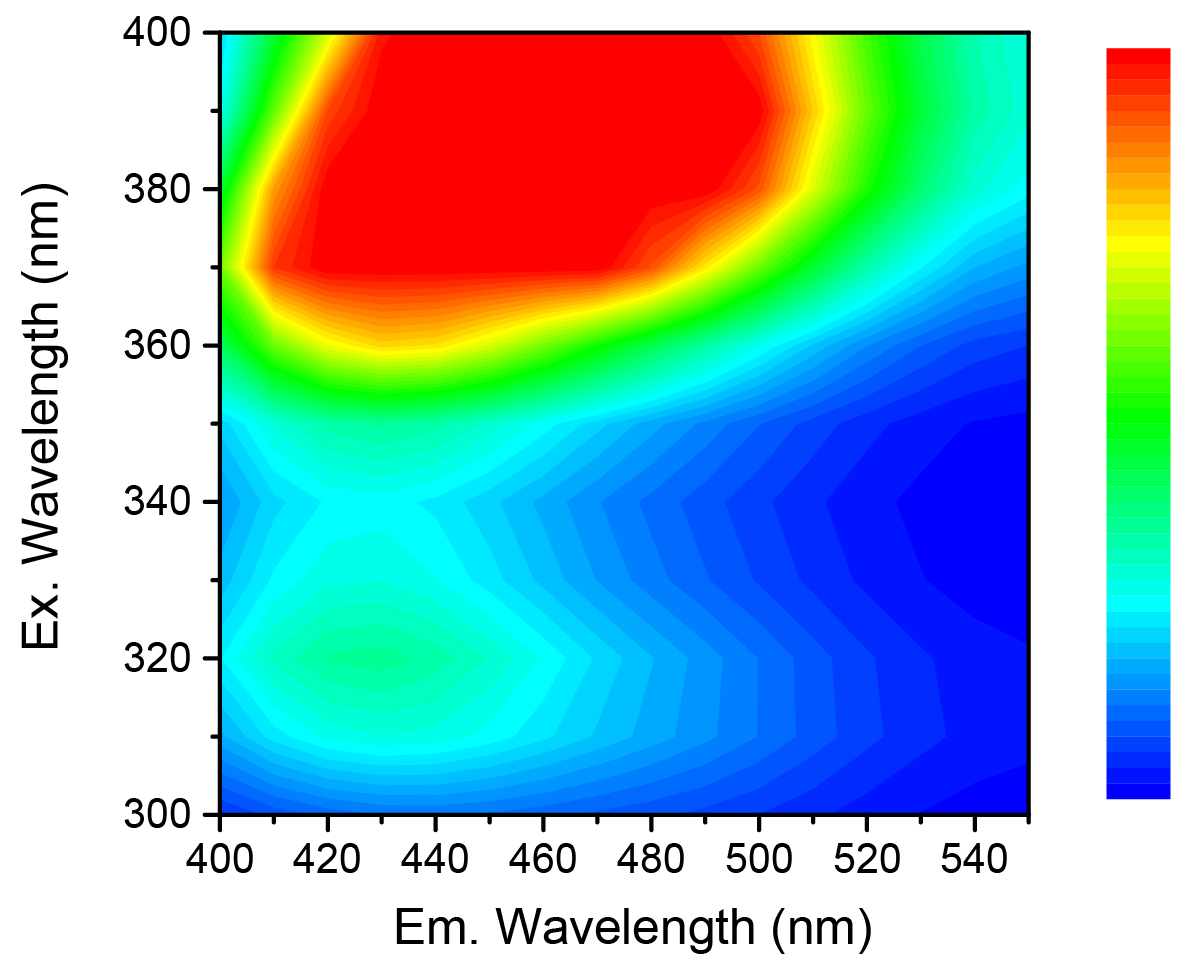


**Fig. S4** The fluorescence excitation-emission contour of the CNDs.


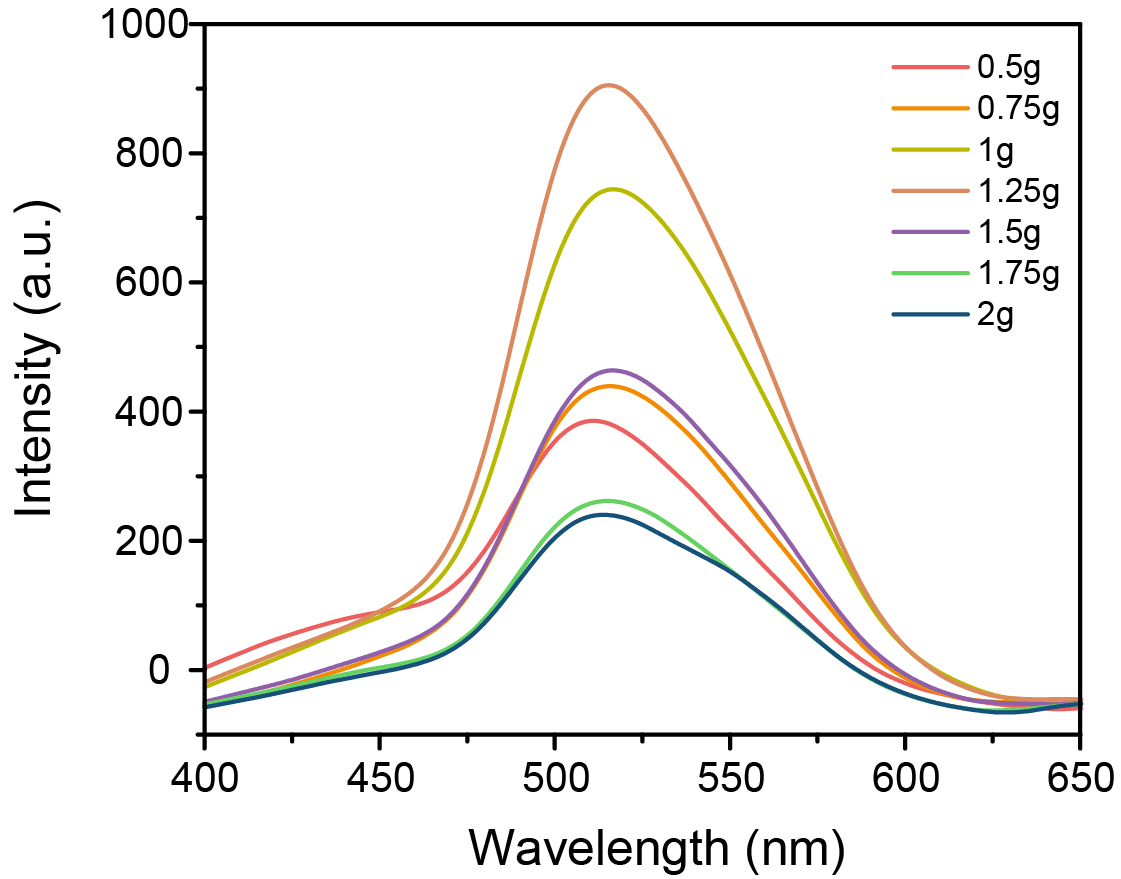


**Fig. S5** The phosphorescence intensity of ultrasound-responsive CNDs with different mass of cyclodextrin.


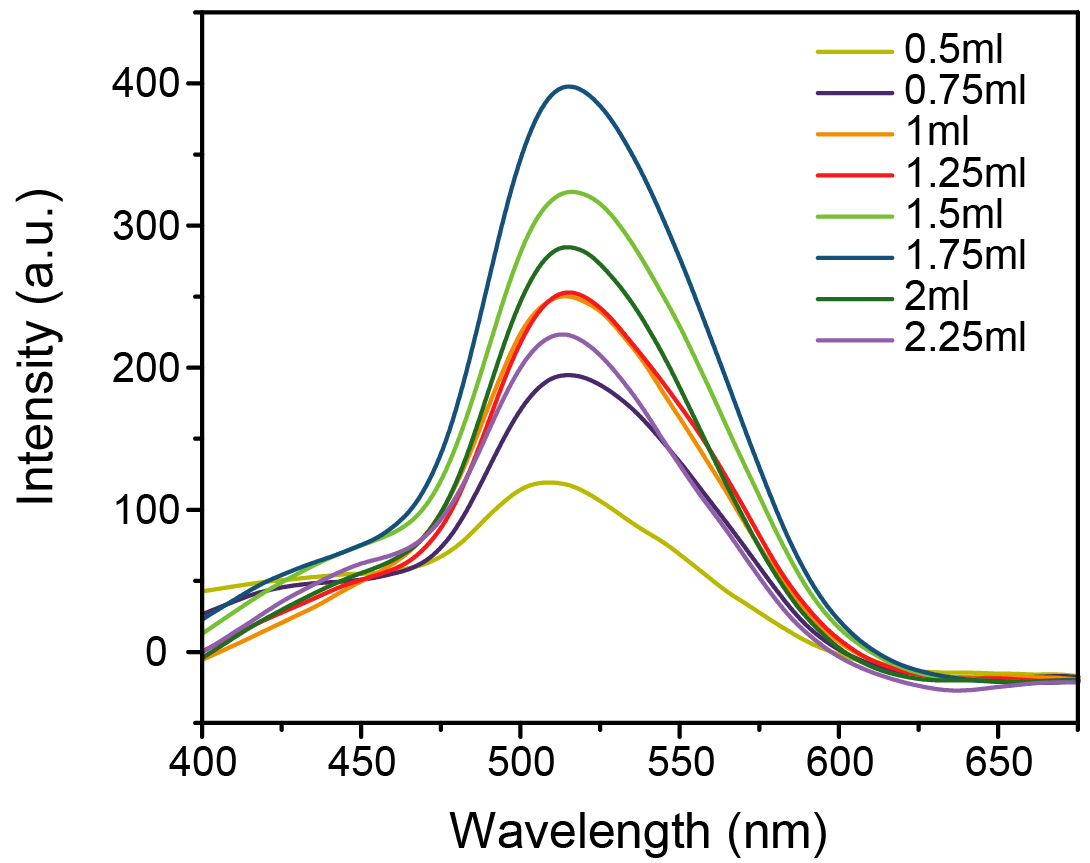


**Fig. S6** The phosphorescence intensity of ultrasound-responsive CNDs with different volume of CNDs.

**
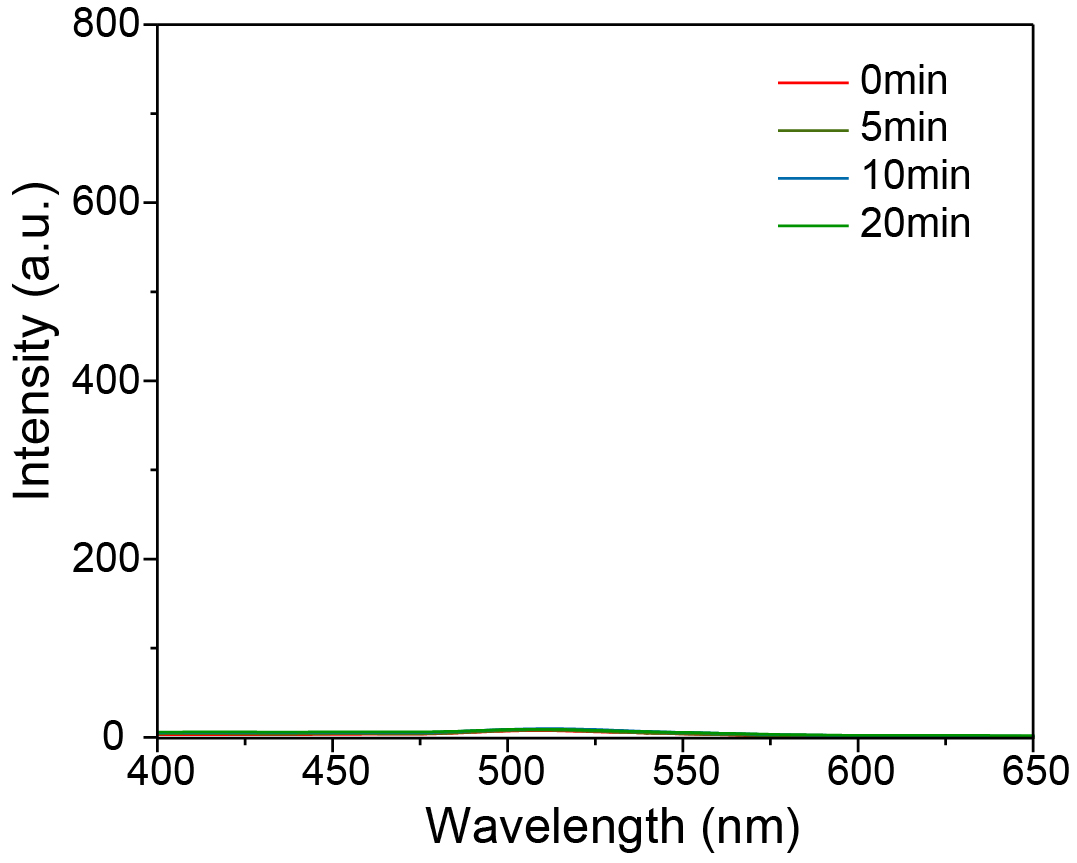
**

**Fig. S7** The mixture of CNDs and cyclodextrin was stirred in water at room temperature without ultrasound treatment.


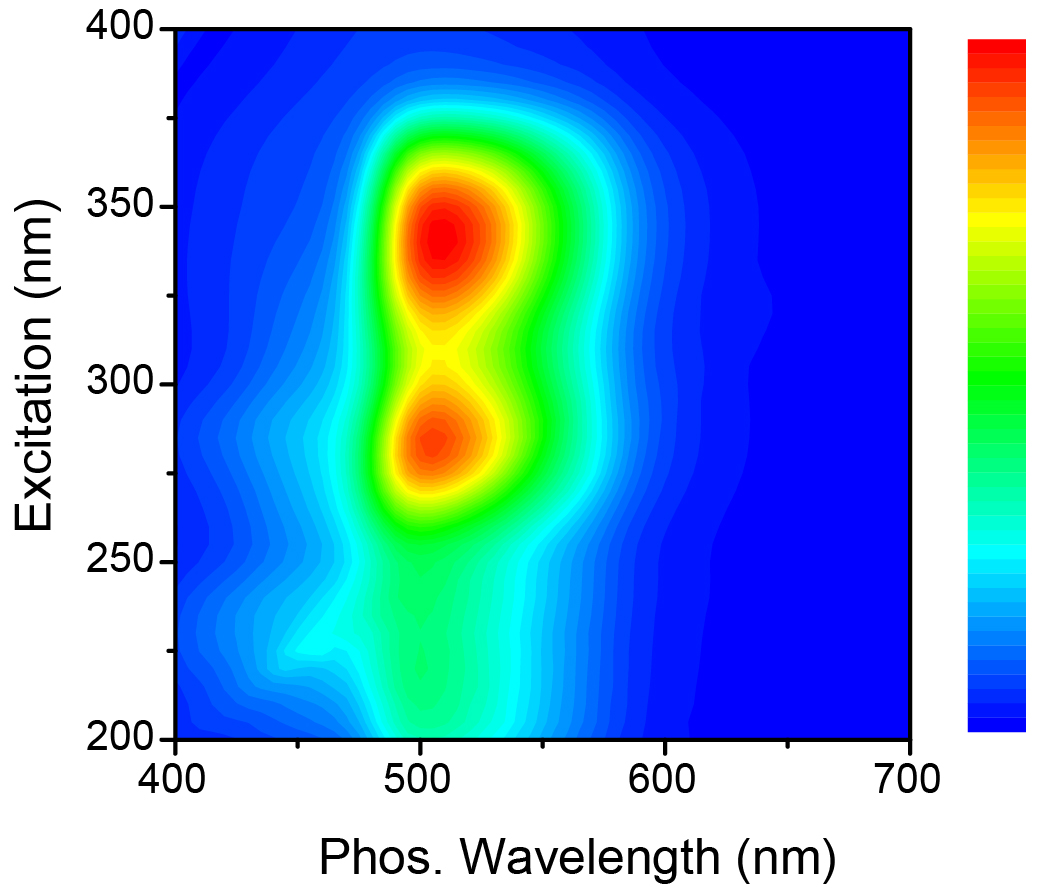


**Fig. S8** The 3D phosphorescence spectrum of ultrasound-responsive CNDs.


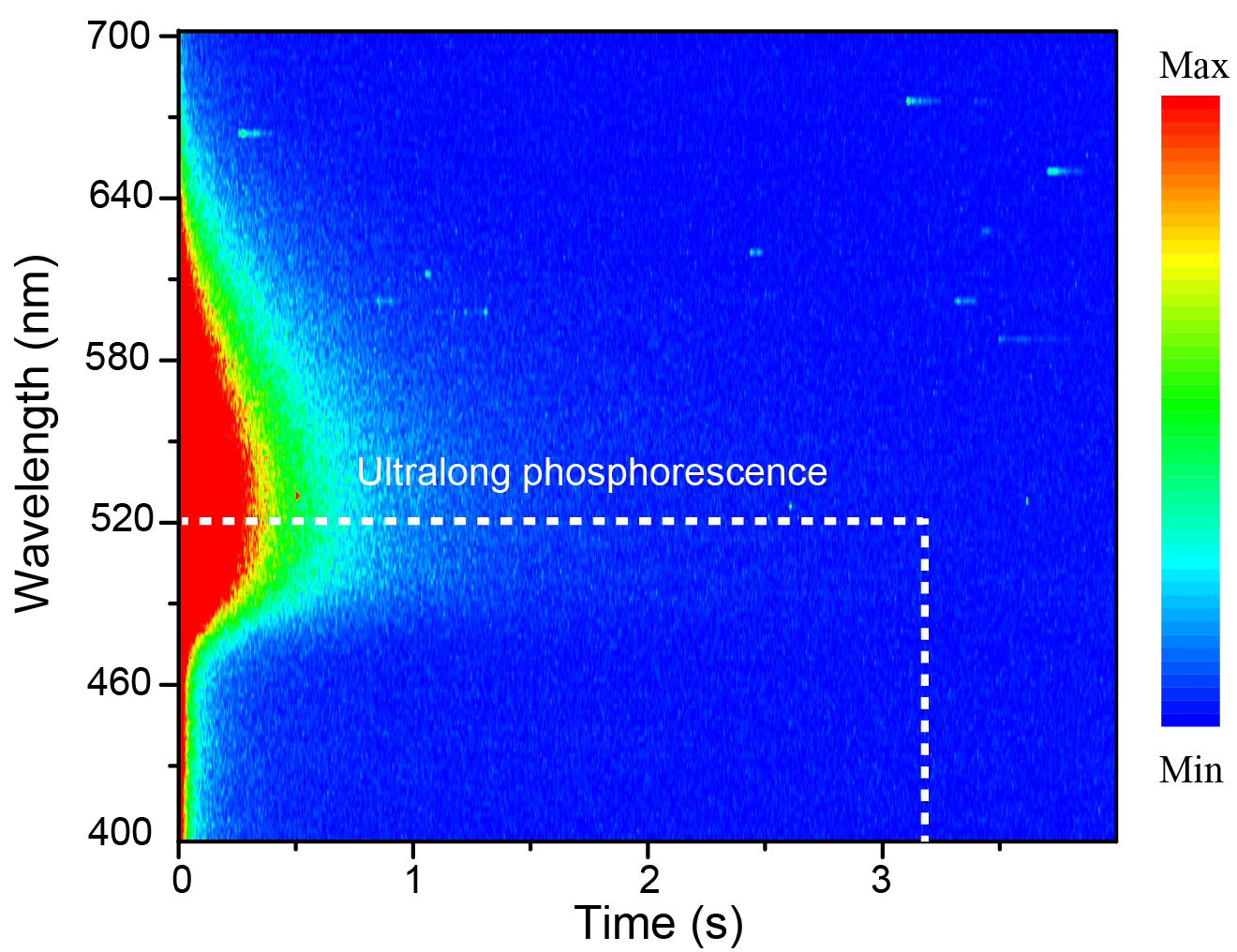


**Fig. S9** Time-resolved emission spectroscopy of ultrasound-responsive CNDs.


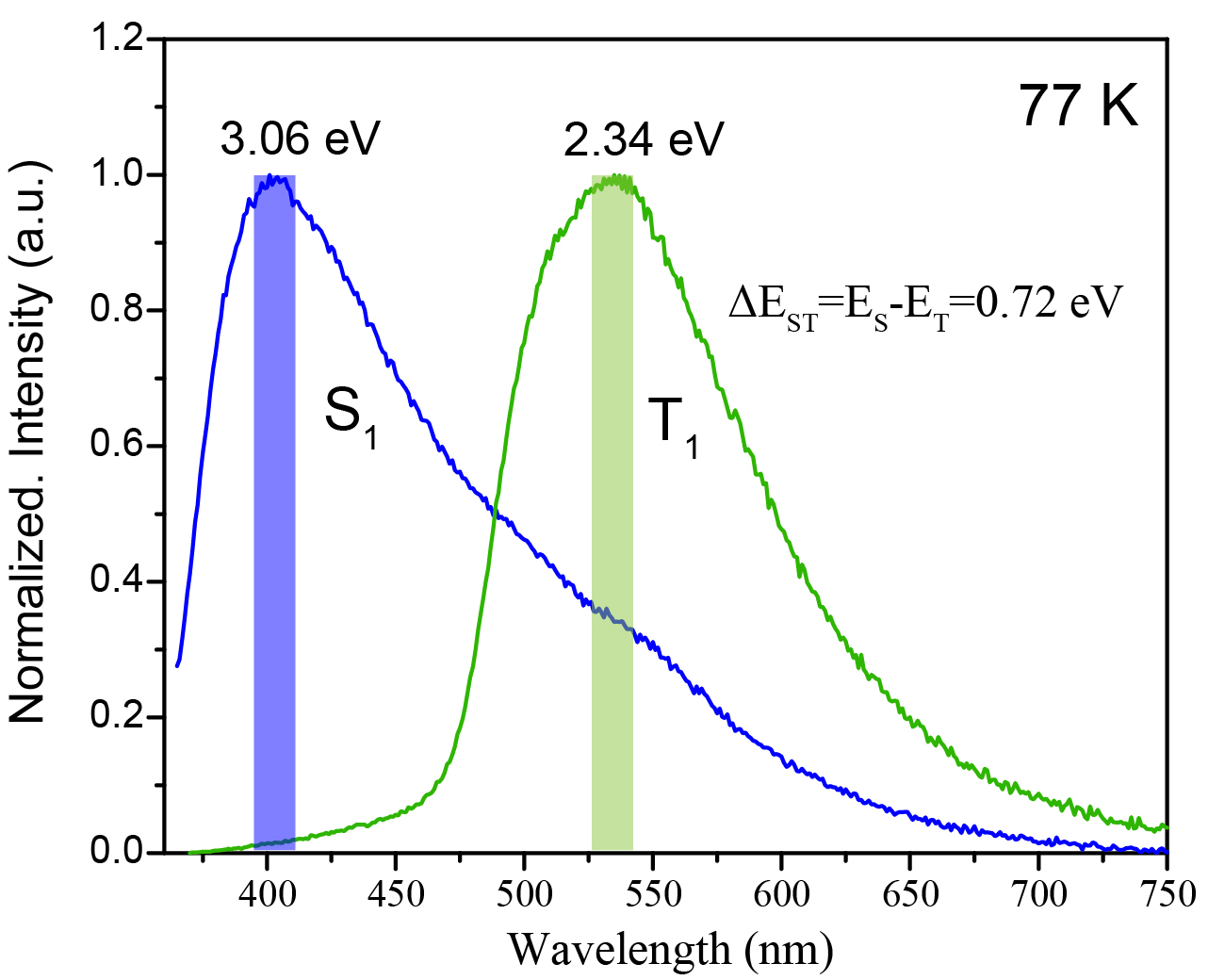


**Fig. S10** The low-temperature fluorescence and phosphorescence spectra of CNDs.


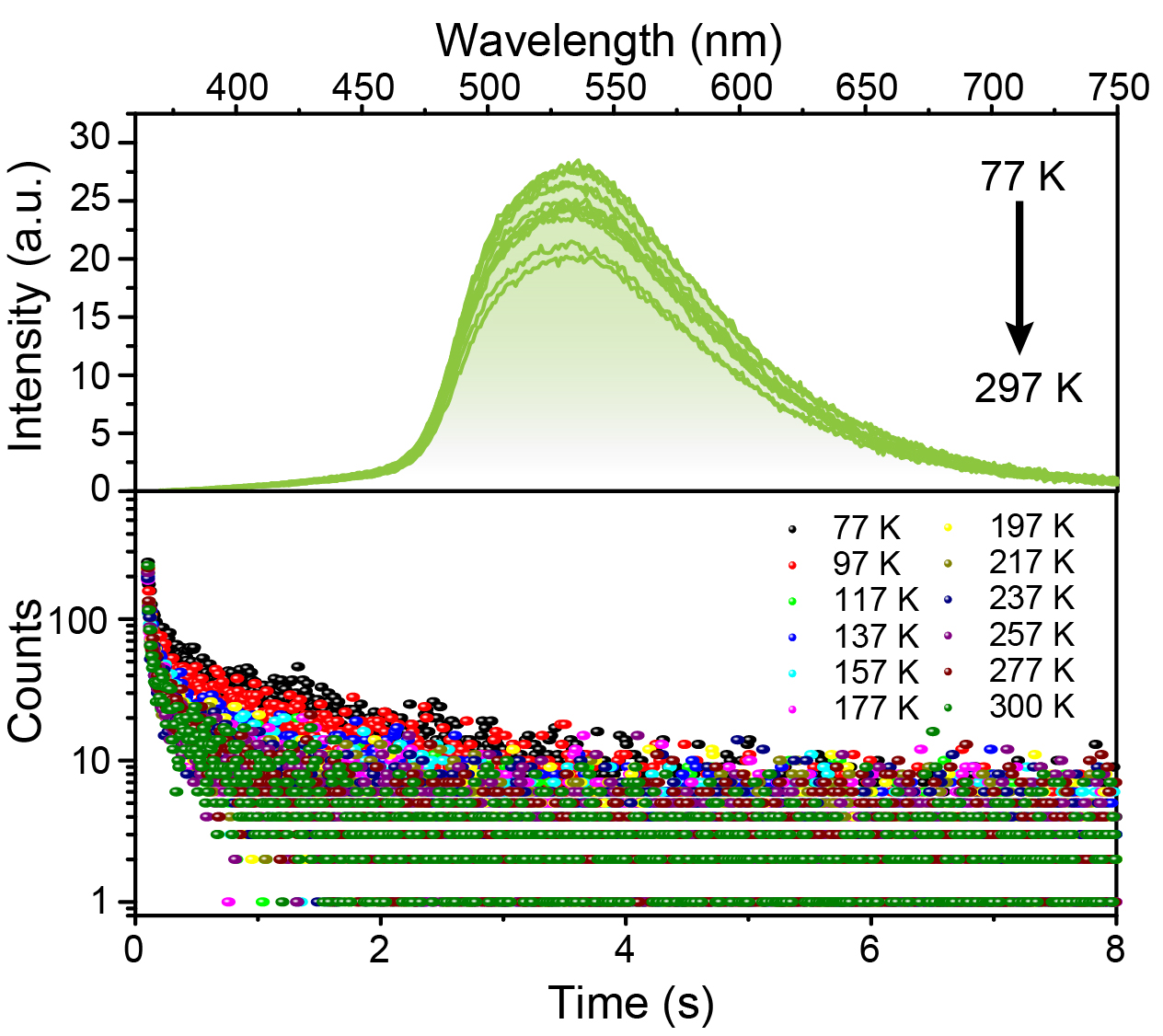


**Fig. S11** The temperature-dependent decay curves and phosphorescence spectra of CNDs.

**
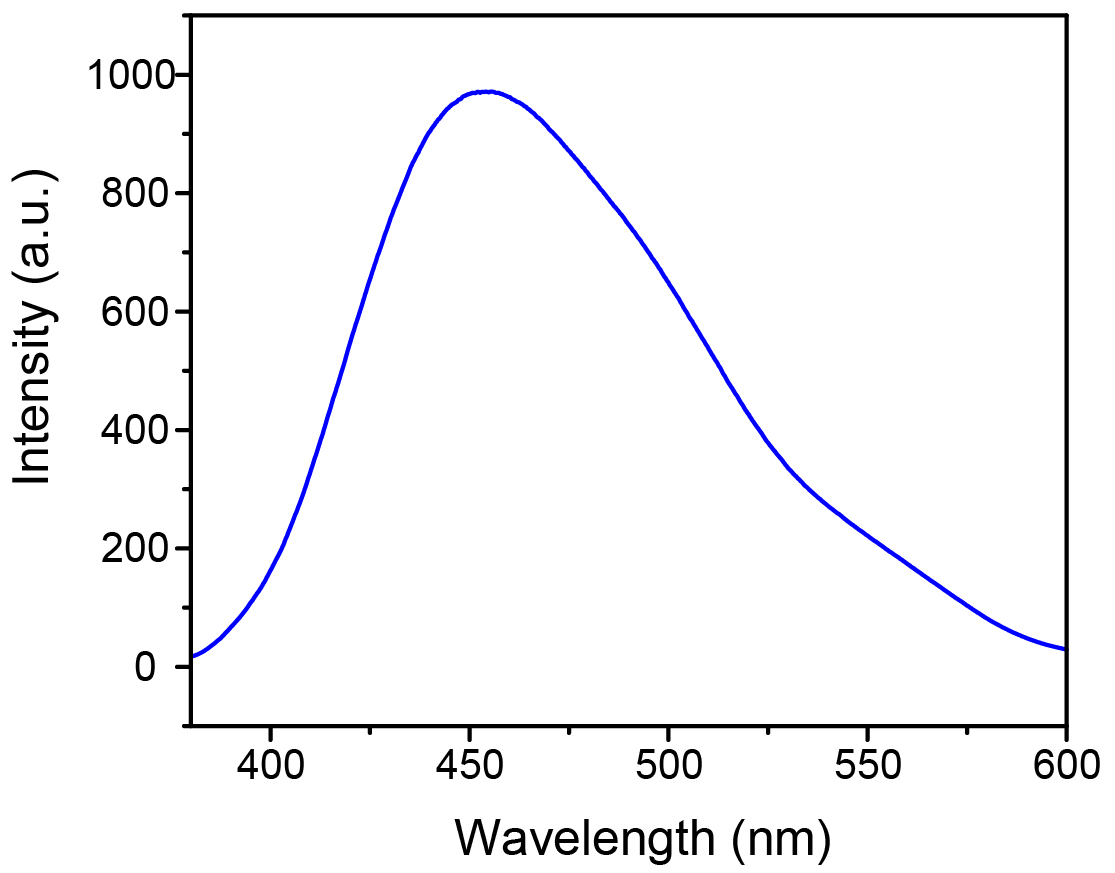
**

**Fig. S12** The fluorescence spectrum of CNDs.

**
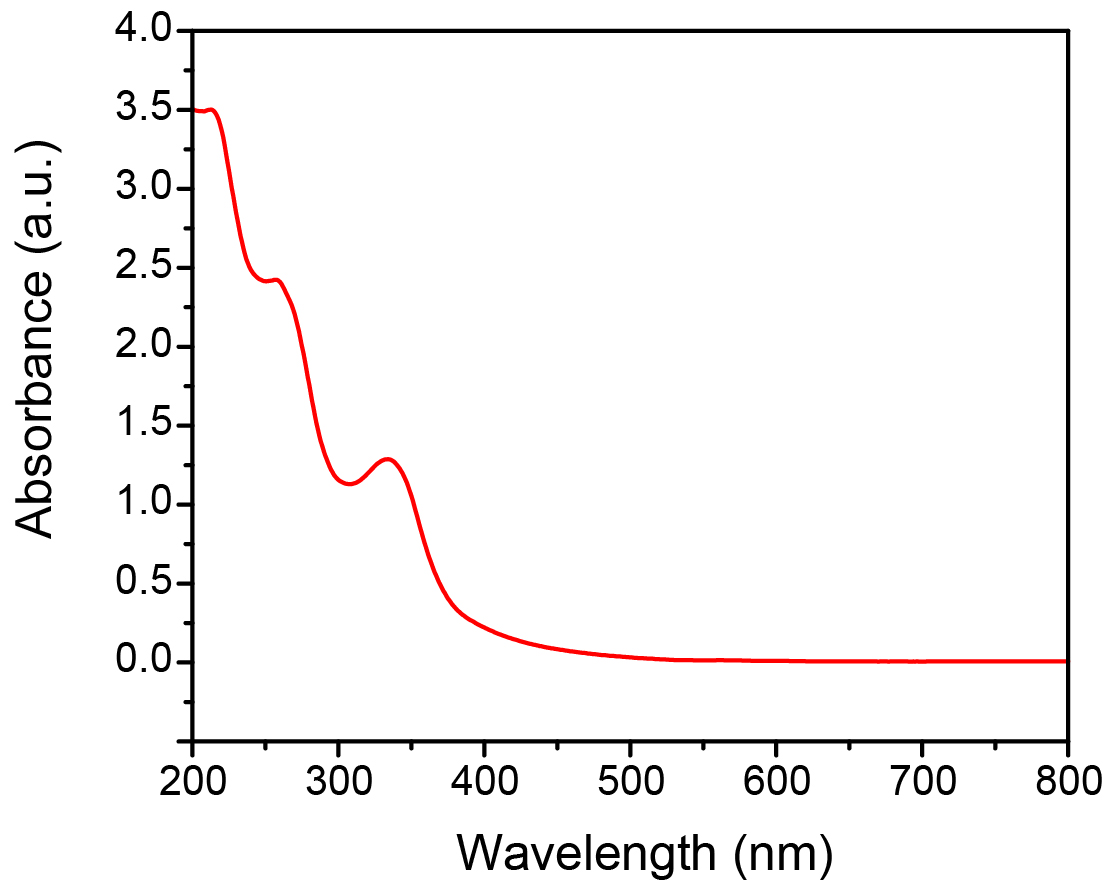
**

**Fig. S13** The absorption spectrum of CNDs.

**
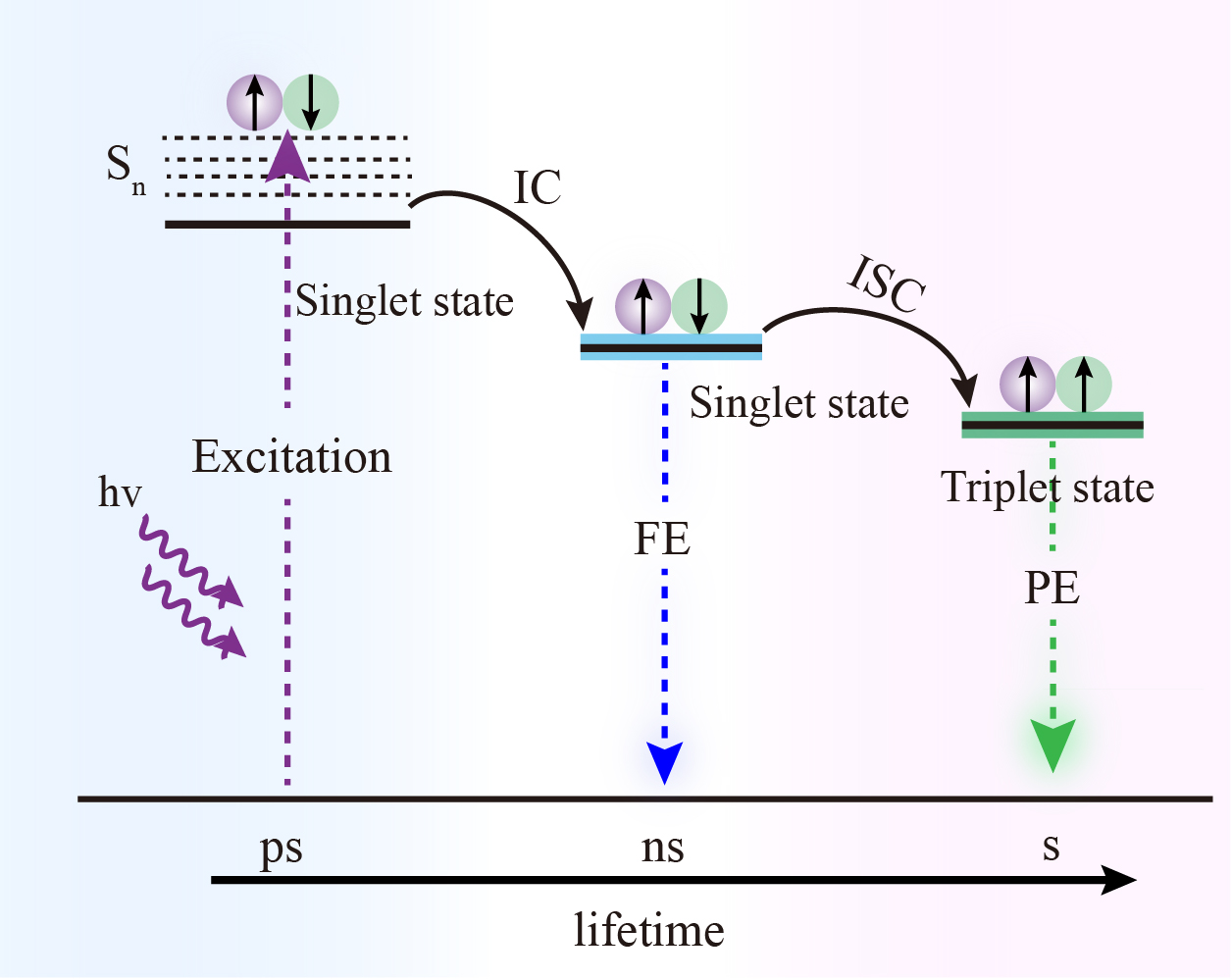
**

**Fig. S14** Proposed mechanism for phosphorescence of CND.

**
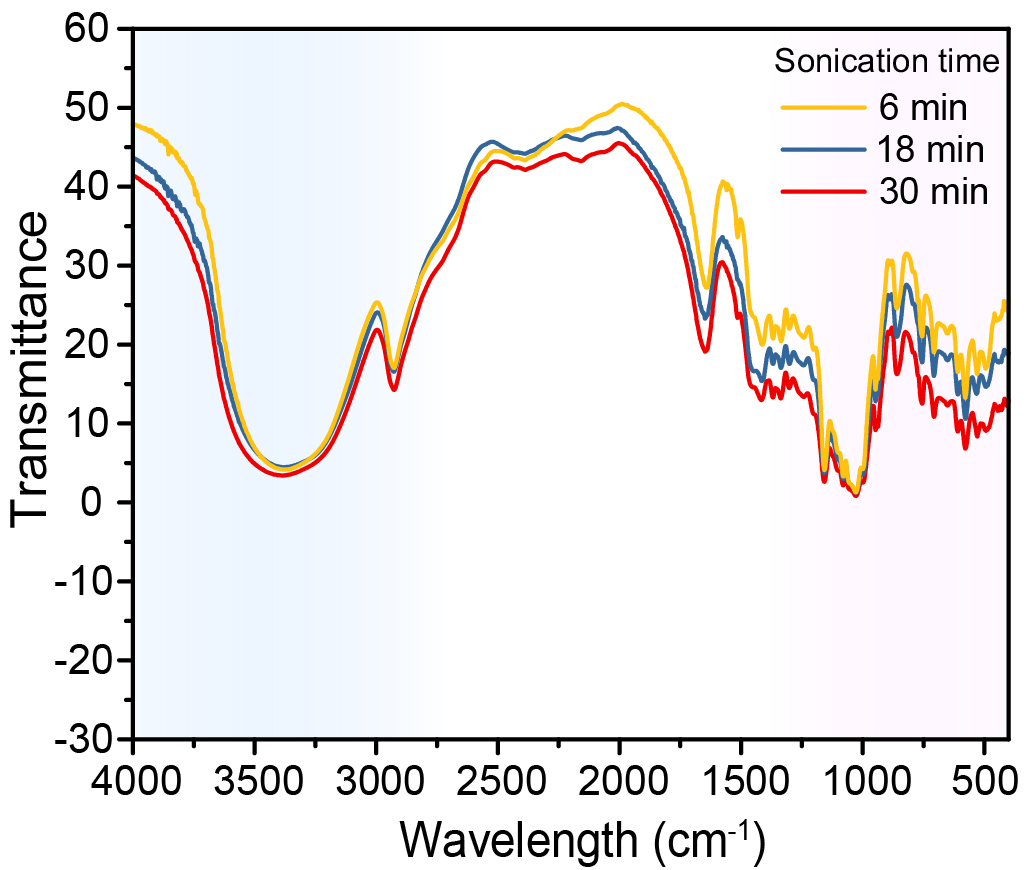
**

**Fig. S15** The Fourier transform infrared spectroscopy spectrum of CNDs.


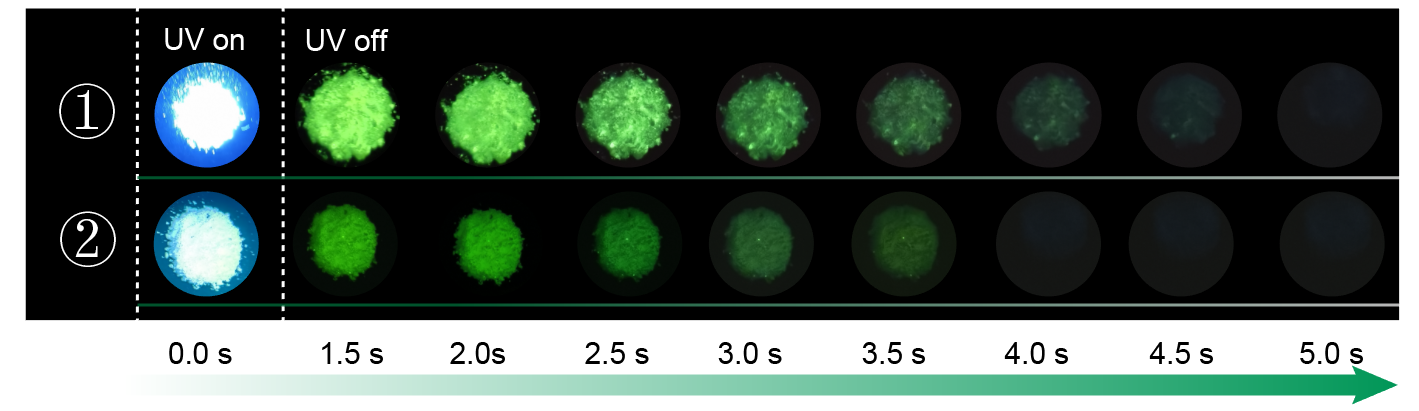


**Fig. S16** The photograph of the CNDs before and after grounding.


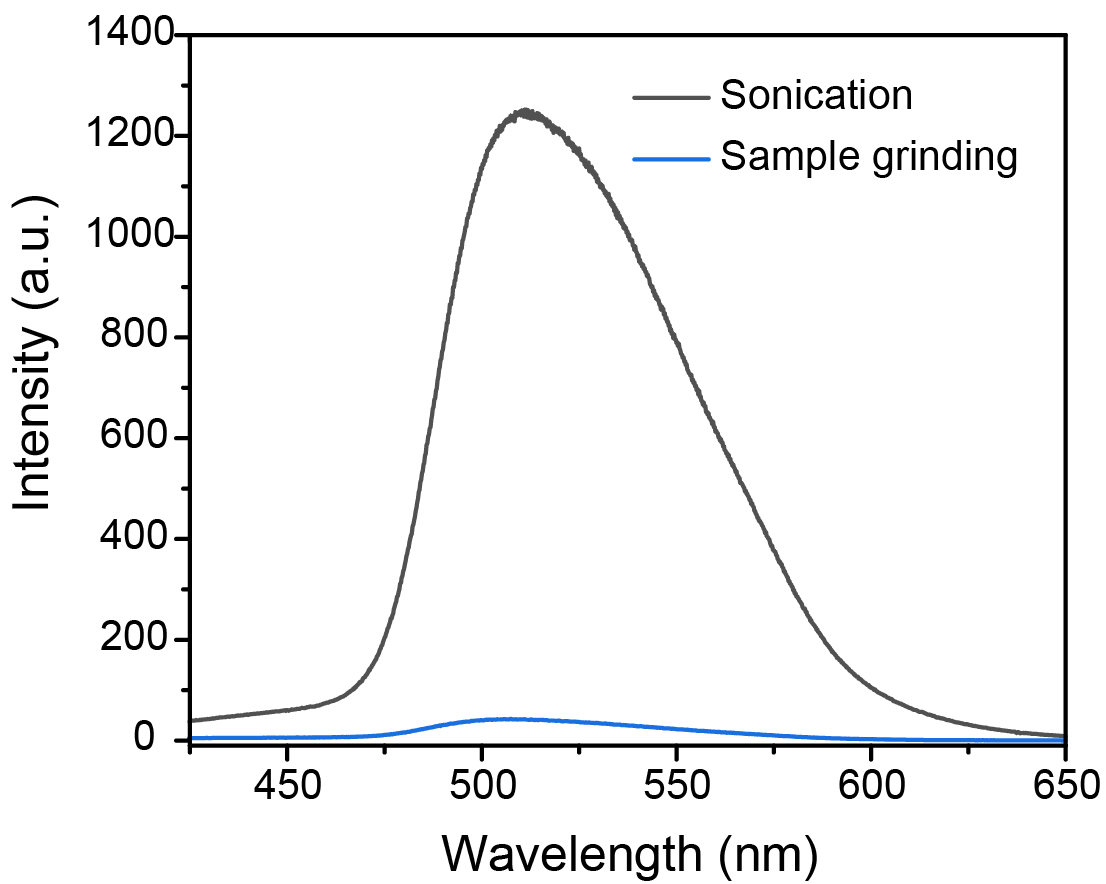


**Fig. S17** The phosphorescence of the grinding CND@CD powder was diminished.


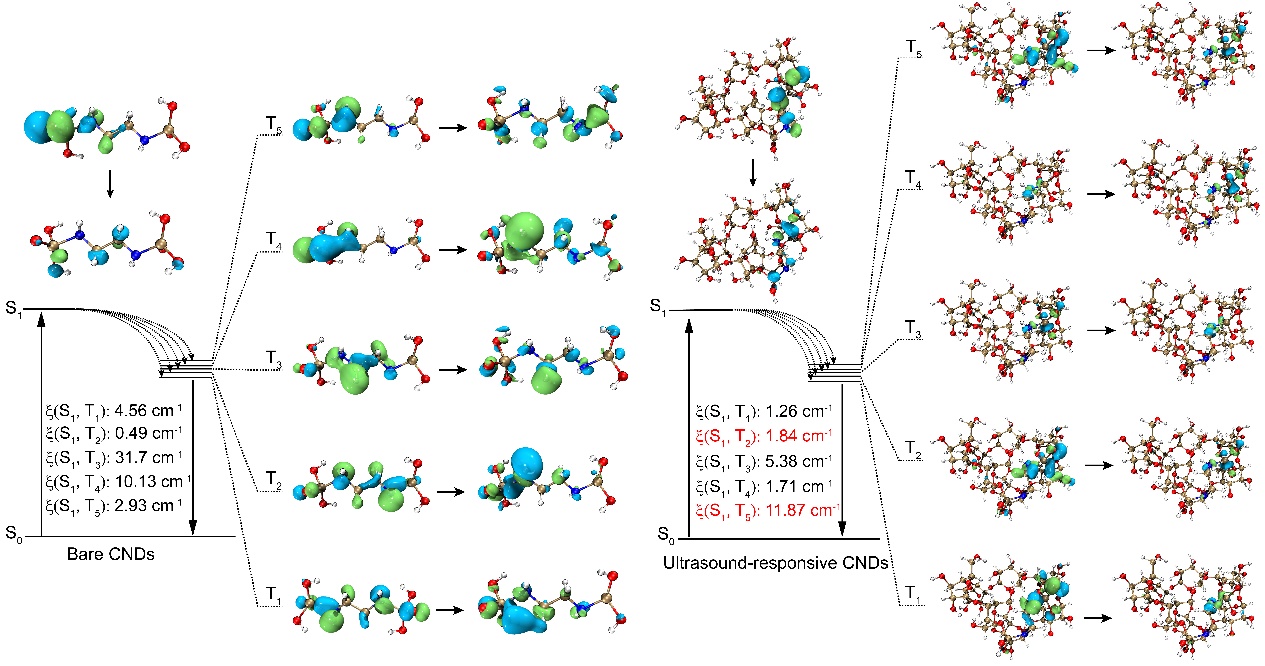


**Fig. S18** Calculated spin-orbit couplings (ξ) and nature transition orbitals for CNDs and ultrasound-responsive CNDs.


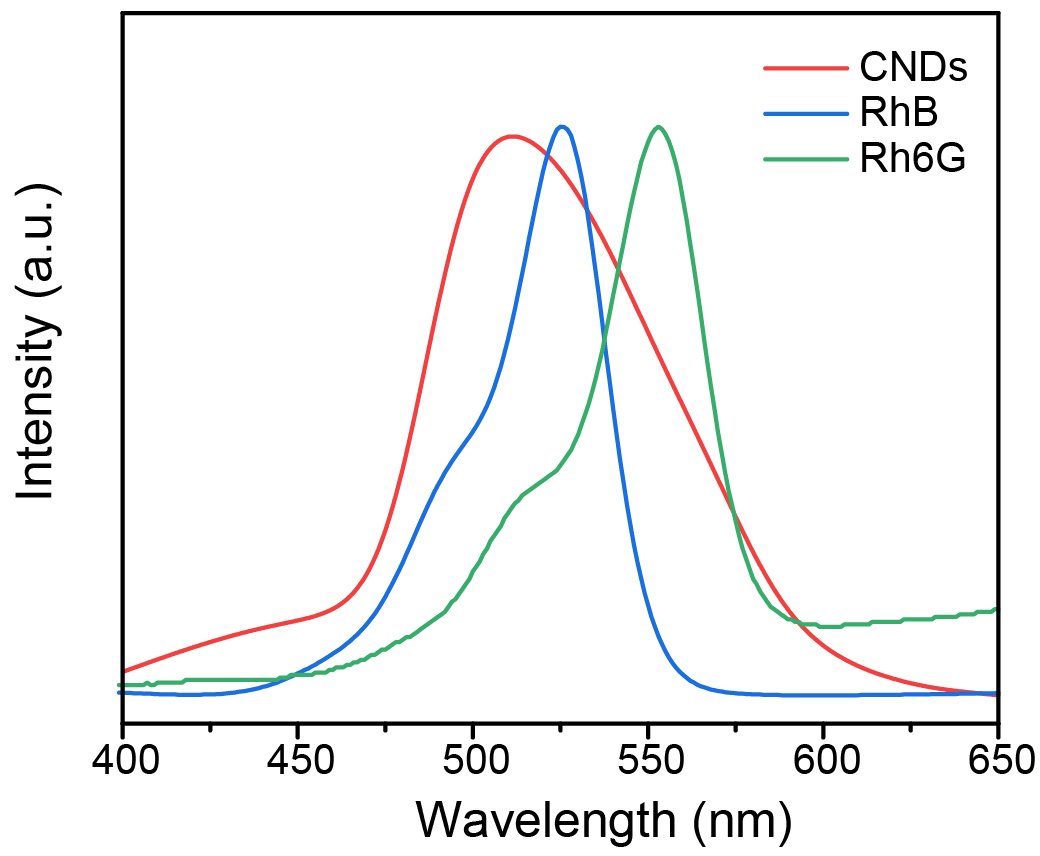


**Fig. S19** Absorption spectra of Rhodamine 6G and Rhodamine B.


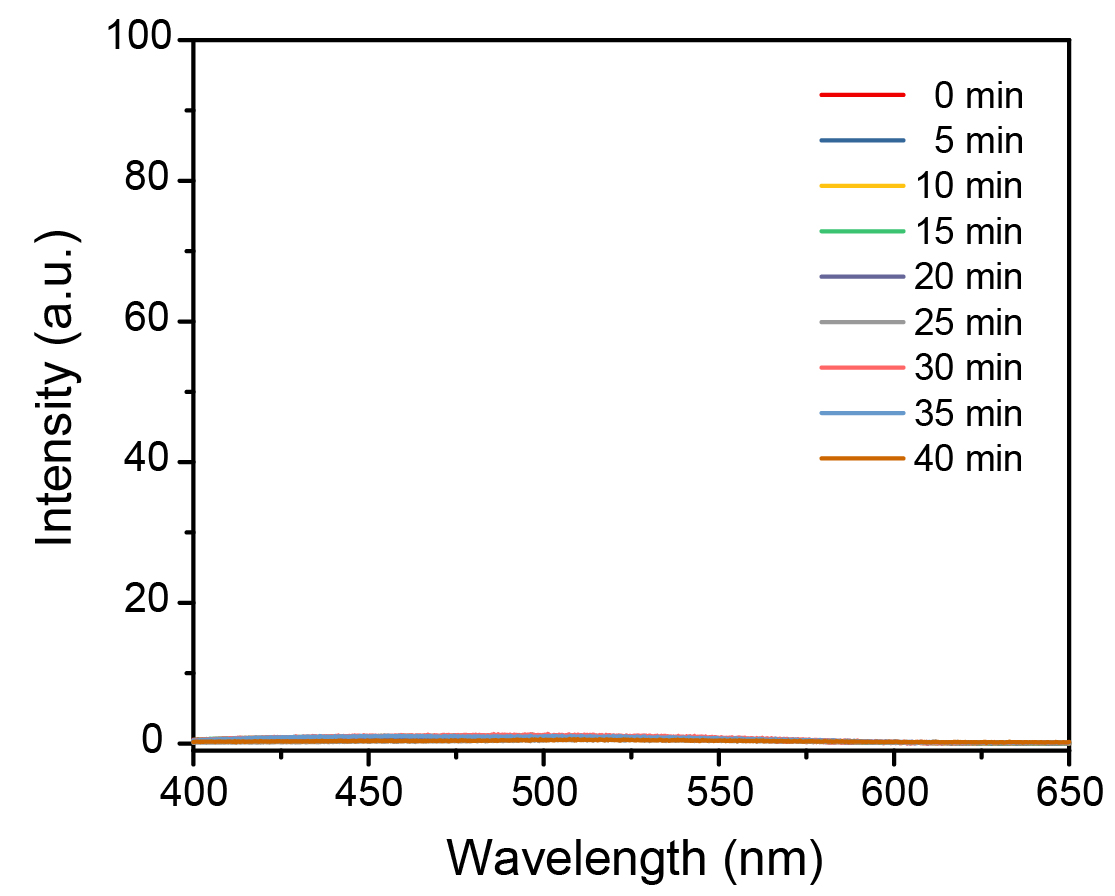


**Fig. S20** Phosphorescence spectra of the CNDs encapsulated by cucurbituril at different ultrasound times.


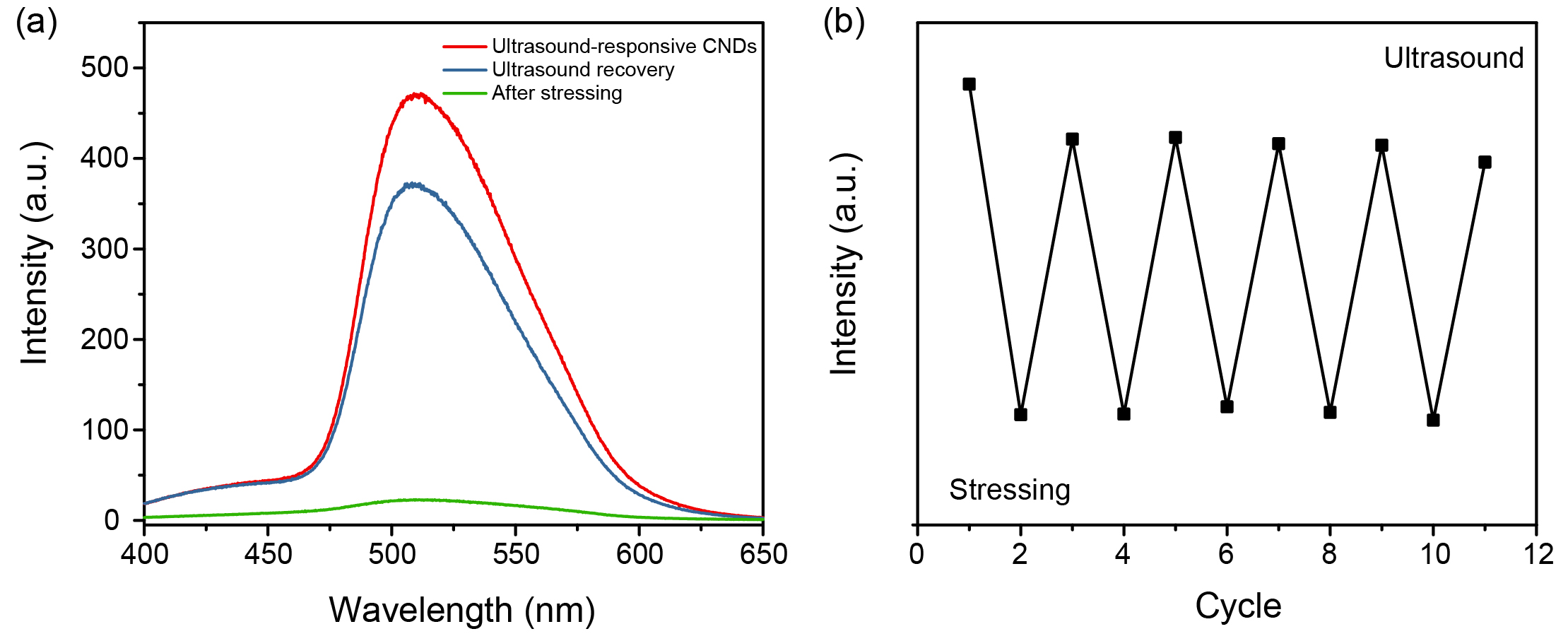


**Fig. S21** **a** Phosphorescence spectra of the ultrasound-responsive CNDs before and after stressing. **b** Phosphorescence intensity of ultrasound-responsive CNDs over five stress-ultrasound recovery cycles.


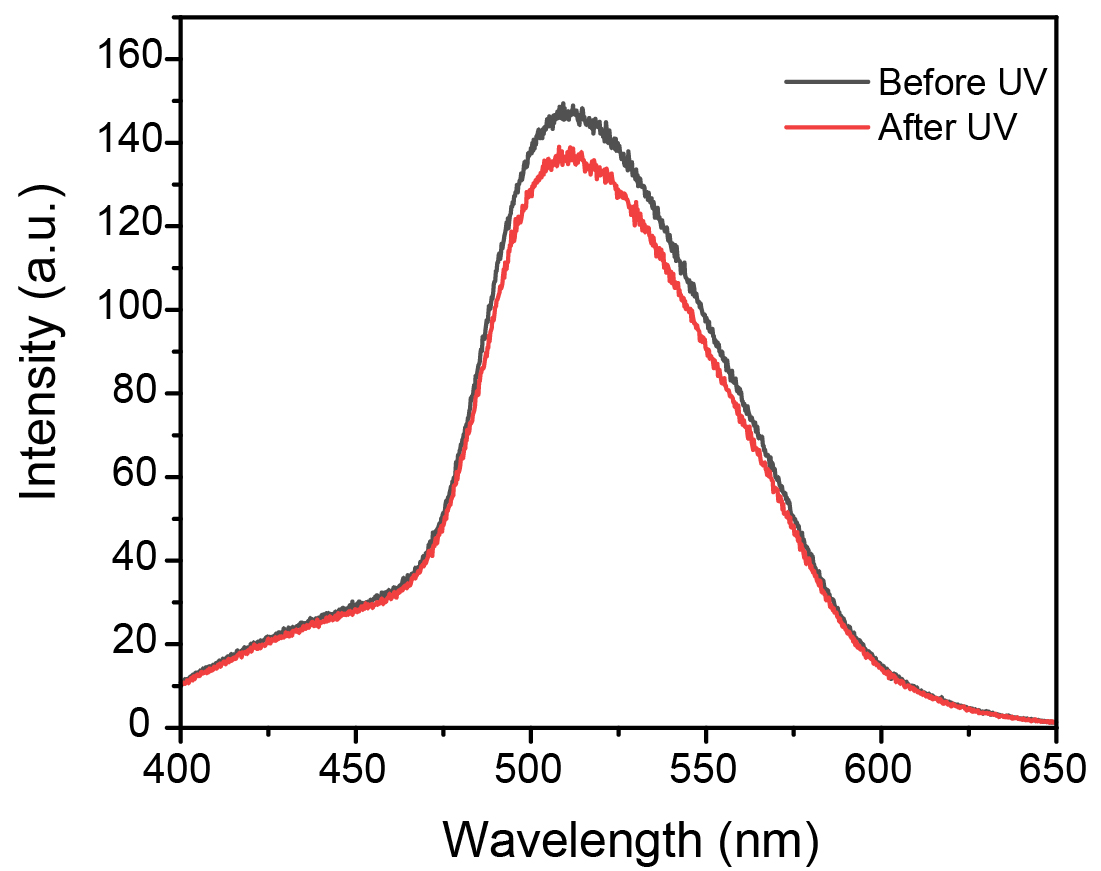


**Fig. S22** Under continuous exposure to UV light for 10 min, the phosphorescence

intensity only showed a slight decrease.


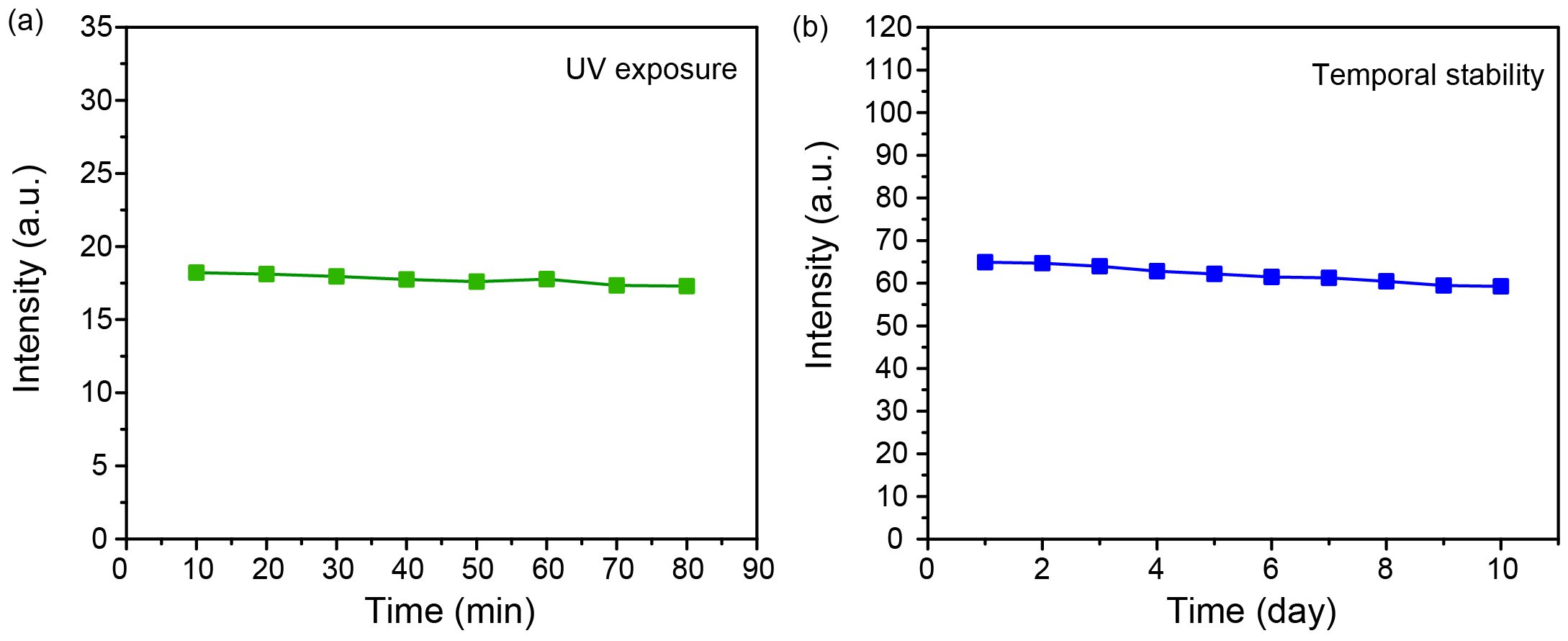


**Fig. S23** **a** Changes in phosphorescence intensity of the CNDs over time after 80 minutes of UV light irradiation. **b** The phosphorescence intensity of the CNDs over days in air.

**Table S1** Dynamic photophysical parameters of the CNDs.

| Ultrasound time (min) | τ_phos_ (s) | φ_phos_ (%) | K_p_ (s^-1^) | k_nr_ (s^-1^) |
| --- | --- | --- | --- | --- |
| 1 | 0.47 | 0.13 | 0.002766 | 2.124894 |
| 5 | 0.54 | 0.97 | 0.017963 | 1.833889 |
| 25 | 0.75 | 3.51 | 0.0468 | 1.286533 |
| 27 | 0.796 | 3.65 | 0.045854 | 1.210427 |
| 35 | 1.208 | 6.49 | 0.053725 | 0.774089 |
| 40 | 1.26 | 6.03 | 0.047857 | 0.745794 |
